# Pantethine ameliorates dilated cardiomyopathy features in PPCS deficiency disorder in patients and cell line models

**DOI:** 10.1038/s43856-025-01017-z

**Published:** 2025-07-31

**Authors:** Fangfang Zhang, Tatjana Dorn, Barbara Gnutti, Yair Anikster, Sarah Kuebler, Rebecca Ahrens-Nicklas, Rachel Gosselin, Shamima Rahman, Ronen Durst, Enrica Zanuttigh, Miriam A. Güra, Christine M. Poch, Anna B. Meier, Karl-Ludwig Laugwitz, Hans-Joachim Schüller, Ana C. Messias, Ody C. Sibon, Dario Finazzi, Alyssa Rippert, Dong Li, Kristen Truxal, Deipanjan Nandi, Brent C. Lampert, Mildrid Yeo, Alice Gardham, Batel Nissan, Smadar Horowitz Cederboim, Alessandra Moretti, Arcangela Iuso

**Affiliations:** 1 Regenerative Medicine in Cardiovascular Diseases, First Department of Medicine, TUM University Hospital, Klinikum Rechts der Isar, Technical University of Munich, School of Medicine and Health, Munich, Germany; 2 Institute of Neurogenomics, Helmholtz Zentrum München, Neuherberg, Germany; 3 Department of Molecular and Translational Medicine, University of Brescia, Brescia, Italy; 4 Metabolic Disease Unit, Edmond and Lily Safra Children’s Hospital, Sheba Medical Center, Tel-Hashomer, Tel Aviv, Israel; 5 School of Medicine, Tel Aviv University, Tel Aviv, Israel; 6 The Children’s Hospital of Philadelphia, Division of Human Genetics, Philadelphia, PA USA; 7 Nationwide Children’s Hospital, Division of Genetic and Genomic Medicine, Columbus, OH USA; 8 UCL Great Ormond Street Institute of Child Health and Great Ormond Street Hospital for Children NHS Foundation Trust, London, UK; 9 Faculty of Medicine, The Hebrew University, Jerusalem, Israel; 10 Department of Cardiology, Hadassah Medical Center, Jerusalem, Israel; 11 Center for Cardiovascular Research (DZHK), Munich Heart Alliance (MHA), Partner Site Munich, Munich, Germany; 12 Center for Functional Genomics of Microbes, Abteilung Molekulare Genetik, Greifswald, Germany; 13 Institute of Structural Biology, Molecular Targets and Therapeutics Center, Helmholtz Zentrum München, Neuherberg, Germany; 14 Bavarian NMR Centre, Department of Bioscience, School of Natural Sciences, Technical University of Munich, Garching, Germany; 15 Department of Biomedical Sciences of Cells and Systems, University Medical Center Groningen, University of Groningen, Groningen, The Netherlands; 16 Nationwide Children’s Hospital, Division of Cardiology, Columbus, OH USA; 17 Division of Cardiology, Department of Medicine, The Ohio State University Wexner Medical Center, Columbus, OH USA; 18 Institute of Human Genetics, Klinikum Rechts der Isar, Technical University of Munich, School of Medicine and Health, Munich, Germany

**Keywords:** Metabolic disorders, Cardiovascular biology

## Abstract

**Background:**

PPCS deficiency disorder (PPCS DD) is an ultra-rare, autosomal recessive form of dilated cardiomyopathy (DCM) caused by pathogenic variants in PPCS, which encodes the enzyme catalyzing the second step in the coenzyme A (CoA) biosynthesis pathway. To date, only six patients worldwide have been identified.

**Methods:**

Whole-exome sequencing was performed to identify pathogenic PPCS variants in affected individuals. Protein stability was assessed by Western blotting. CoA levels were quantified using a microplate-based assay in patient-derived fibroblasts, cardiac progenitor cells, and cardiomyocytes. Functional evaluation of cardiac cells and engineered heart patches was conducted to investigate contractile performance and arrhythmogenicity. Pantethine was tested as a potential therapeutic agent both in vitro and through long-term clinical follow-up in patients.

**Results:**

Causative PPCS variants are identified in six individuals with DCM and variable associated features, including neuromuscular and neurological symptoms. Identified variants lead to reduced PPCS protein stability and decreased cellular CoA levels. Cardiac cells exhibit impaired contractility and arrhythmias, which are partially rescued by pantethine treatment. Clinically, patients receiving pantethine show sustained improvement over time.

**Conclusions:**

Our study expands the genetic and clinical spectrum of PPCS deficiency disorder, identifying six new cases with diverse phenotypes. Functional investigations reveal reduced CoA levels and dysfunction in patient-derived cardiac cells. Pantethine treatment shows promise in partially rescuing DCM phenotypes, both in vitro and in patients. However, complete reversal may require early intervention. These findings underscore the importance of timely diagnosis and treatment in PPCS DD. Future research should focus on optimizing pantethine supplementation and exploring additional therapies to enhance CoA levels and cardiac function in affected individuals.

## Abstract (web summary)

Zhang, Dorn, Gnutti et al. identify pathogenic PPCS variants in people with PPCS deficiency disorder. Cardiac cells exhibit impaired contractility and arrhythmias, which is partially rescued by pantethine treatment.

## Plain language summary

PPCS deficiency disorder is an extremely rare inherited disease that causes heart muscle weakness (dilated cardiomyopathy) and other symptoms. It results from changes in a gene involved in making coenzyme A (CoA), a vital molecule for cell energy. This study identified six new patients with the condition and investigated how these gene changes affect heart function. Researchers used patient cells and lab-grown heart tissues to study the disease and tested pantethine, a compound that helps increase CoA levels. They found that pantethine improved heart cell function and showed positive effects in treated patients. These results highlight the importance of early diagnosis and treatment. In the future, therapies such as pantethine could offer hope for improving heart health in affected individuals.

## Introduction

Coenzyme A (CoA) is an essential metabolite for over 100 metabolic reactions. Comparative genomics provided evidence that cells produce CoA de novo from pantothenate (vitamin B5) through five consecutive enzymatic steps performed by four enzymes^1^. In order: pantothenate kinase (PANK), phosphopantothenoylcysteine synthetase (PPCS), phosphopantothenoylcysteine decarboxylase (PPCDC), and the bifunctional coenzyme A synthase (COASY) (Fig. S1). Pathogenic variants in PPCS and PPCDC lead to PPCS and PPCDC deficiency disorders (hereafter indicated with PPCS DD and PPCDC DD, respectively), two ultra-rare diseases presenting with dilated cardiomyopathy (DCM), often fatal in early childhood, with variable neuromuscular and extra-cardiac manifestation, and no apparent neurodegeneration^2–4^. In contrast, pathogenic variants in PANK2 and COASY are associated with the rare neurological diseases Pantothenate Kinase-Associated Neurodegeneration (PKAN) (4) and COASY protein-associated neurodegeneration (COPAN) (5), respectively, characterized by progressive neurodegeneration with brain iron accumulation (NBIA) and no cardiac symptoms (Fig. S1). So far, the coexistence of neurological impairment and cardiac problems has been observed only in PPCS-deficient fruit flies^2,3,5^. Regardless of the clinical presentations, patients with PKAN, PPCD DD, PPCDC DD and COPAN have a short life expectancy and face the devastating effects of these conditions daily^2,6,7^.

Since PANK, PPCS, PPCDC and COASY are all required for CoA de novo biosynthesis^2,4,5,7,8^, boosting CoA levels has been suggested as a sensible therapeutic approach in PKAN, PPCD DD, PPCDC DD and COPAN^9^.

Providing elevated doses of pantothenic or CoA are two ineffective approaches. In one case because patients are incapacitated to enzymatically convert pantothenic acid to CoA, and in the other case because CoA is degraded to pantothenic acid in the gut. On the contrary, nutritional interventions with pantethine and 4’-P-pantetheine, two derivatives of pantothenic acid, are expected to increase CoA levels in PKAN, PPCS and PPCDC DDs because they enter the pathway downstream of PPCDC, thus bypassing the enzymatic blockages of PANK, PPCS and PPCDC^9^ (Fig. S1).

In animal models of PKAN and PPCS DD, pantethine alleviated many of the disease phenotypes^2,10–13^. In patients affected by PKAN pantethine improved quality of life without improving substantially their motor functions (Trial no. ChiCTR1900021076)^14^. In two siblings affected by PPCS DD pantethine improved exertional dyspnea and ventricular ejection fraction in one case and stabilized heart conditions in the other^2^. However, considering the limited duration of the treatment at the time of reporting and the anecdotal number of treated patients, it is hard to draw conclusions on the efficacy of pantethine in PPCS DD.

4’-P-pantetheine has shown promising rescue effects in in vivo fruitfly and mouse models of PKAN^15^, and is currently being tested as a treatment for PKAN in two clinical trials^16,17^. Like pantethine, 4’-P-pantetheine is reported to be well-tolerated^18^. Since 4’-P-pantetheine is more stable than pantethine to the degradation by vanins/pantetheinases in plasma^19^, 4’-P-pantetheine is expected to be more beneficial than pantethine in the treatment of PKAN, PPCS and PPCDC DDs^9^. However, neither in vitro nor in vivo data have been generated on the utilization of 4’-P-pantetheine to treat PPCS and PPCDC DDs.

In the current project, we expanded the phenotypic spectrum of PPCS DD, by reporting on the identification of six additional patients carrying three new pathogenic variants on PPCS. Moreover, we generated 2D and 3D models of PPCS DD using patient-derived fibroblasts and induced pluripotent stem cells (iPSCs) and defined the onset of biochemical and functional alterations during cardiac lineage commitment and maturation. Besides, we compared the efficacy of pantethine and 4’-P-pantetheine in boosting CoA levels in fibroblast of PPCS DD cases, showed that pantethine supplementation reverts specific features of DCM phenotypes in cardiomyocytes, and report on the outcome of a long-term pantethine treatment in two previously reported cases and in two newly identified cases.

## Methods

### Patient identification and recruitment

Patients for this study were identified through a comprehensive review of medical records at participating tertiary care centers specializing in rare metabolic disorders and cardiology. We focused on individuals with confirmed genetic mutations in the PPCS gene, which is associated with the condition under investigation. Potential participants were initially screened based on their clinical presentation, family history, and genetic test results. Once identified, these patients and their families were approached by their treating physicians, who provided information about the study. We explained the study’s objectives, procedures, potential risks, and benefits in detail. A list of study participants, clinical features and information on pantethine intervention is provided in Table S1.

### Ethical approval

Informed consent was obtained from all adult participants, while for minors, assent was obtained along with parental consent. Consent was also obtained from all participating families for the inclusion of identifiable information in this publication. The study was conducted in accordance with the principles embodied in the Declaration of Helsinki and approved by the Institutional Review Boards of the Children’s Hospital of Philadelphia (16-013278), Sheba Medical Center (SMC615819) and Hadassah University Hospital (0151-20-HMO) ensuring ethical standards were maintained throughout the recruitment process. The use of pantethine for the patients was approved as a compassionate treatment (29 C) by the Israeli Ministry of Health.

Generation of iPSCs from patient fibroblasts and differentiation to cardiac cells was approved by the Ethics Committee of the Technical University of Munich (2022-220-S-NP).

Fresh pig hearts for the generation of porcine extracellular matrix were obtained from Munich slaughterhouse as a waste from a licensed abattoir producing meat for consumption operating under strict government protocols. Therefore, the ethical approval of fresh pig hearts was not required.

### Inclusion and diversity statement

Our inclusion criteria encompassed patients of all ages and genders with genetically confirmed PPCS mutations, regardless of their current clinical status or previous treatments. This approach allowed us to capture a diverse range of disease presentations and progression stages. We also ensured diversity in experimental samples by selecting various cell lines. The author list of this paper includes contributors from the research location who participated in data collection, design, analysis, and/or interpretation of the work.

### Treatment of patients with pantethine

Pantethine was supplemented to various patients at different doses. Patient F1.II commenced pantethine treatment at a dose of 450 mg/day (17 mg/kg/day divided BID, Jarrow Formulas) which was later increased to 900 mg/day. Patient F5:IV.1 is taking 600 mg/day (10 mg/kg/day). The two affected individuals from family 6, F6:IV.1 and F6:IV.4, already reported in a previous study^2^ continued oral treatment with pantethine supplementation for four (Patient F6:IV.1 died of COVID-19) and six years, respectively. The daily dose was approximately 15 mg/kg weight from different manufacturers (currently, Jarrow, Pure Encapsulation, Coenzyme-A Technologies).

### Structural biology prediction analysis

Structural files of the human (PDB: 1P9O)^20^ and the orthologous S. cerevisiae (PDB: 6AIK)^21^ PPCS were downloaded from the protein data bank (PDB). Structural models of Y78H and R106P mutants were computed using FoldX5.0 suite^22^ using the human PPCS as a starting model (1P9O) after minimization of the structures. Stability analyses were performed using FoldX5.0 suite^22^. Images of the structures were created using UCSF Chimera 1.15^23^.

### Cell culturing of primary fibroblasts

Fibroblasts, established from skin biopsies of the patients (Table S2) or purchased by a commercial vendor (NHDF, #CC-2509, Lonza) were cultured in high glucose Dulbecco’s Modified Eagle Medium (Life Technologies, Carlsbad, CA, USA, 41966029) supplemented with 10% fetal bovine serum (Life Technologies, 10270106), 50 U/mL penicillin/streptomycin (Life Technologies, 15070063), and 200 μM uridine (Sigma, St. Louis, MI, USA, U3750), at 37 °C in a 5% CO_2_ humidified atmosphere. All cell lines tested negative for mycoplasma contamination using the MycoAlert Mycoplasma detection kit (Lonza, LT07-118), according to the manufacturer’s instructions.

### Western blotting

Analysis of PPCS protein level was performed on whole cellular lysate under denaturing conditions, and proteins were separated by sodium dodecyl sulfate-polyacrylamide gel electrophoresis (SDS-PAGE; Lonza, PAGEr EX Gels). Gels were blotted onto PVDF membrane (GE-Healthcare) for subsequent incubation with primary antibodies and probing with appropriate secondary antibodies. Chemiluminescence was documented on a Fusion FX7 system (Peqlab). The following antibodies were used for western blotting: rabbit anti-PPCS (ab140626, 1:2,000) purchased from Abcam, mouse anti-Tubulin (T5168, 1:15,000) and mouse anti-Actin (A5441, 1:15,000) purchased from Sigma-Aldrich. HRP-conjugated secondary antibodies for western blot (111-036-045; 115-036-062) were obtained from Jackson Immunoresearch Laboratories (USA) (1:15,000).

### iPSC line generation, cell culture, and cardiac differentiation

The iPSC lines were generated from 2 PPCS patients (Table S2) and 2 healthy subjects by reprogramming fibroblasts or peripheral blood mononuclear cells using the CytoTune^TM^-iPS 2.0 Sendai Reprogramming Kit (Thermo Fisher Scientific, A34546), as previously described^24,25^. The iPSC lines generated from the patients were registered as HMGUi003-A and MRIi028-A, while those from the control subjects were registered as MRIi001-A and MRIi003-A. All iPSC lines were fully characterized^26^. One clone per each control and patient lines were used.

The iPSCs were cultured on Geltrex-coated (Thermo Fisher Scientific, A14133-02) tissue culture plates (Falcon, 353001) in Essential 8 medium (Thermo Fisher Scientific, A1517001) containing 0.5% Penicillin/Streptomycin (Thermo Fisher Scientific, 15140122) under standard culture conditions (37 °C, 5% CO_2_). Daily medium changes were performed, and cells were passaged every 4-5 days using 0.5 mM EDTA (Thermo Fisher Scientific, AM9260G). To promote survival after passaging, the medium was supplemented with 10 μM Thiazovivin (Sigma-Aldrich, SML1045) for 24 hours (h).

To induce differentiation of iPSCs into cardiomyocytes (iPSC-derived CMs), a Wnt/β-catenin signaling based-protocol described by Foo et al. was applied^27^. Briefly, iPSCs were dissociated with 0.5 mM EDTA and seeded on Geltrex-coated 24-well plates (Nunclon, 142475) at a density of 1 × 10^5^ cells per well. Once the cells reached 95% confluence after 3-4 days, cardiomyocyte differentiation was induced on day 0 by changing to RPMI1640 (Thermo Fisher Scientific, 21875034) containing B27 insulin minus (Thermo Fisher Scientific, A1895601) (defined as basal cardiac differentiation medium) supplemented with 1 µM CHIR-98014 (Selleckchem, S2745). On day 1, the medium was replaced with basal cardiac differentiation medium. On day 3, medium was replaced with basal cardiac differentiation medium supplemented with 2 µM Wnt-C59 (Selleckchem, S7037). From day 5 onwards, the medium was replaced with RPMI1640 medium containing B27 insulin plus. Typically, spontaneous beating was observed on days 7-8.

For long-term maintenance of cardiomyocytes, beating areas were dissected manually and transferred to fibronectin-coated plates at around day 20–24 of differentiation. The isolated cell clumps (named as explants) were maintained in EB2 medium consisting of DMEM/F-12 (Thermo Fisher Scientific, 11320033) supplemented with 2% fetal bovine serum (FBS, Thermo Fisher Scientific, 16141079), 1% non-essential amino acids (Thermo Fisher Scientific, 11140050), 0.5% Penicillin-Streptomycin (Thermo Fisher Scientific, 15140122), 1% L-glutamine (Thermo Fisher Scientific, 25030081) and 0.1 mM β-mercaptoethanol (Sigma-Aldrich, M6250).

### Dissociation of iPSC-derived CMs

The iPSC-derived CMs were harvested at 15 (d15) and 60 days (d60) of cardiac differentiation. For dissociation, the iPSC-derived CMs were treated with papain (Worthington Biochemical Corporation, LS003124) following a protocol described by Fischer and colleagues, with some modifications^28^. Briefly, d15 iPSC-derived CMs were rinsed twice with 2 mM EDTA in DPBS without Ca^2+^/Mg^2+^ (Sigma-Aldrich, D8537) and treated with a solution containing 20 U/mL papain and 1 mM L-cysteine (Sigma-Aldrich, C6852) in DPBS at 37 °C for 30 min. To stop the reaction, a solution containing 1 mg/mL trypsin inhibitor (Sigma-Aldrich, T9253) and 40 µg/mL DNAse I (Sigma-Aldrich, DN25) in DPBS was added in a 1:1 ratio. For d60 iPSC-derived CMs, the explants were dissected and incubated with papain solution under shaking at 37 °C for 30 min. Afterwards, the trypsin inhibitor solution was used to stop the reaction. The single cells were then collected either for further cultivation in 2D and 3D or for immunofluorescence and flow cytometry analysis.

For assessment of calcium transients and sarcomere organization, d53 iPSC-derived CMs cultured were dissociated and plated on fibronectin-coated plates in EB20 (DMEM/F12 containing 20% heat-inactivated FBS, 1x MEM nonessential amino acids, 1x penicillin/streptomycin, 0.1 mM β-mercaptoethanol and 2 mM L-glutamine). Next day, medium was replaced by EB2 (DMEM/F12 containing heat-inactivated 2% FBS, 1x MEM nonessential amino acids, 1x penicillin/streptomycin, 0.1 mM β-mercaptoethanol and 2 mM L-glutamine), 100 µM pantethine was applied in EB2 medium two days after reseeding and medium change was performed every other day. On day 5 after initial pantethine treatment, CMs were either used for calcium measurements or fixed for immunostaining,

### Porcine extracellular matrix (ECM) generation and 3D cultivation of iPSC-derived CMs

Porcine extracellular matrix (ECM) was prepared as previously described^24,26^. Briefly, porcine myocardial tissue obtained from left mid-ventricular transmural sections was immediately placed in PBS (Thermo Fisher Scientific, 10010015). The tissue was then embedded in 4% agarose in PBS and sliced into 300 µm thick sections using a vibratome (VT1200S, Leica Biosystems, Germany). Next, the heart slices were decellularized overnight in lysis buffer (10 mM Tris, 0.1% EDTA, pH 7.4) with agitation on an orbital shaker at room temperature (RT), followed by incubation in 0.5% SDS solution with agitation on an orbital shaker at RT for at least 6 h. Samples were then washed three times with PBS and incubated in 50% FBS in PBS overnight at 4 °C. Then, the ECM slices were washed with PBS and stored in PBS containing 1% Penicillin-Streptomycin up to three weeks at 4 °C. The sex of the pig hearts used for ECM preparation was not known.

For 3D cultivation of iPSC-derived CMs, small plastic triangles were attached to heart ECM slices with tissue adhesive (Histoacryl, B. Braun 69390) in the direction of the muscle fiber. iPSC-derived CMs were dissociated at day 15 of differentiation into single cells using papain and reseeded onto ECM slices placed in cell culture plate inserts (Millipore, PICM03050) in 6 well plates. Underneath the inserts, 1 mL EB2 medium was added, and medium was exchanged daily. 7 days after reseeding, the ECM slices with iPSC-derived CMs were anchored in biomimetic culture chambers (InVitroSys) and subjected to physiological preload of 1 mN and stimulation at 1 Hz (50 mA pulse current, 1 ms pulse duration). The slices were kept in EB2 medium (DMEM/F12 containing 2% FBS, 1x MEM nonessential amino acids, 1x penicillin/streptomycin, 0.1 mM β-mercaptoethanol and 2 mM L-glutamine), which was replaced every other day, on a rocker plate (60 rpm, 15 °C tilt angle) placed in an incubator set at 37 °C, 5% CO_2_, 20% O_2_ and 80% humidity. A continuous readout of contraction force was obtained via the biomimetic chamber^26,28–30^. For the effective refractory period (ERP) measurements, the patches were paced with a programmed train of S1 stimuli at a fixed cyclic length, followed by a S2 premature stimulus with gradual shortening of the S1-S2 interval. For assessment of force-frequency-relationship, the patches were subjected to programmed increasing pacing frequencies and contraction force was recorded. Contractility data were analyzed by LabChart Reader software.

### Calcium imaging of CMs in 2D and in 3D patches

Calcium imaging was performed as previously described^26^, with some modifications. In brief, iPSC-derived CMs cultured in 2D were loaded with 1 μM Fluo-4-AM (Thermo Fisher Scientific, F14201) in Tyrode’s solution (135 mM NaCl, 5.4 mM KCl, 1 mM MgCl_2_, 10 mM glucose, 1.8 mM CaCl_2_ and 10 mM HEPES, pH7.35) at 37 °C for 30 min. To perform calcium imaging on iPSC-derived CMs cultured in 3D, 300 μm-thick patches were incubated with 1 μM Fluo-4-AM in Tyrode’s solution containing 0.01% Pluronic F-68 (Gibco, 24040-032) for 50 min at 37 °C. Subsequently, the solution on 2D iPSC-derived CMs and 3D heart patches was replaced with Tyrode’s solution and incubated for 30 min at 37 °C to allow for de-esterification of the dye. Imaging was then carried out at 100 fps using an inverted epifluorescence microscope (DMI6000B, Leica Microsystems) equipped with a Zyla V sCMOS camera (Andor Technology, Germany). Field stimulation electrodes (RC-37FS, Warner Instruments) connected to a stimulus generator (HSE Stimulator P, Hugo-Sachs Elektronik) were used to perform pacing, which consisted in providing depolarizing pulses at the indicated frequencies. The fluorescence of single cells relative to background regions was quantified in ImageJ (National Institutes of Health), and subsequent analysis was conducted in RStudio (RStudio: Integrated Development for R. RStudio, Inc., Boston, MA) using custom-written scripts. After subtraction of the background fluorescence, the time course of Fluo-4 fluorescence was expressed as arbitrary units (A.U.) The amplitude of calcium transients was calculated by subtracting basal fluorescence (F_0_) from the peak fluorescence (F), and then normalizing F-F_0_ to the baseline value F_0_ [ΔF/F_0_ = (F-F_0_)/F_0_].

### Freezing and cryosections of iPSC-derived CM patches in 3D cultivation

For freezing and cryosections, iPSC-derived CM patches were washed with DPBS^+/+^ and fixed with 4% paraformaldehyde (PFA; Sigma-Aldrich, 158127) for 20 min at RT. The samples were then washed once for 5 min with DPBS^+/+^ before being placed in a sucrose gradient (Sigma-Aldrich, S9378-1KG) in DPBS^+/+^ of 7.5%, 15% and 30% for 20 min each at RT. To prepare for embedding, the cryomolds (Tissue-Tek, 4566) were fully covered with 50% O.C.T. (Tissue-Tek, 4583) in 30% sucrose without introducing bubbles. The patches were then carefully transferred to the cryomold with as little carryover of 30% sucrose solution as possible. The patches were then positioned under a microscope, taking care to ensure they were as flat as possible within the cryomold. To freeze the samples, 2-methyl-butane (Sigma-Aldrich, M32631) was placed in a liquid nitrogen bath and cooled down for 2-3 min. The cryomolds were then immersed in the cooled 2-methyl-butane for 1-2 min until frozen. After freezing, the samples were stored at −80 °C until ready to be cut into 10 μm-thick cryosections. The cryosections were cut and transferred onto poly-L-lysine slides (Thermo Fisher Scientific, J2800AMNT) using a Microm HM 560 cryostat (Thermo Fisher Scientific, Germany). The slides were left to dry for 30 min at RT before being stored at −80 °C for long-term storage.

### Transient transfection of HeLa cells and immunocytochemistry

One day before transfection, mycoplasma-free HeLa cells (#CCL-2, ATCC; no cell authentication performed after purchase) were seeded at a density of 2 × 10^4^ cells per imaging dish (Miltenyi Biotec, 130098284) in high glucose Dulbecco’s Modified Eagle Medium without antibiotics. Transfection was performed using 1 µg DNA (PPCS, ENST00000372561.4, in pcDNA6.2-GFP) Lipofectamine 2000 (Invitrogen) according to the manufacturer’s protocol.

24 h post transfection, cells were fixed with 4% PFA for 10 min at RT, washed twice with PBS and permeabilized using a 0.1% NP-40 solution in PBS for 10 min at RT. Primary and secondary antibodies were diluted in a 2% BSA, 0.1% NP-40 solution in PBS at a 1:200 and 1:500 ratio, respectively. Cells were incubated with rabbit polyclonal anti-TOM20 (Santa Cruz, sc-11415) and mouse monoclonal anti-GAPDH (ab110305, Abcam) overnight at 4 °C and then for 1 h with AF647 anti-rabbit (ab150079, Abcam) and AF568 anti-mouse (Invitrogen, A11004). Nuclei were stained with DAPI in the Prolong gold Antifade Mountant (Invitrogen, P36931), and images were acquired by confocal microscopy (Leica TCS SP5).

### Immunofluorescence analysis of iPSC-derived CMs in 2D and 3D patches

For immunofluorescence staining of cells cultured in 2D, cells were fixed with 4% PFA for 10 min at RT and washed three times with DPBS^+/+^. Afterwards, cells were permeabilized with 0.25% Triton X-100 (Sigma-Aldrich, X100) for 15 min at RT before washing three times with DPBS^+/+^, followed by incubation with 10% FBS in DPBS^+/+^ containing 0.1% TritonX-100 (PBST) for 1 h at RT. To perform immunofluorescence staining on 3D patches, the slides were taken out from −80 °C freezer and allowed to dry for 30 min at RT. The slides were then fixed with 4% PFA for 10 min at RT, followed by 3 times washing with DPBS^+/+^ for 5 min each. Afterwards, the slides were permeabilized with 0.25% Triton X-100 for 15 min at RT and subsequently incubated with 10% FBS in PBST for 1 h at RT.

Primary antibodies against cardiac Troponin (cTnT, Thermo Fischer Scientific, MA5-12960; Abcam, ab92546), α-actinin (Sigma-Aldrich, A7811), ISL1 (DSHB, 39.4D5) and PPCS (Thermo Fisher Scientific, PA5-95630) were incubated in 1% FBS in PBST overnight at 4 °C. After washing 5 times for 5 min with PBST, they were incubated with appropriate secondary antibodies and Phalloidin (Thermo Fisher Scientific, A12379) in PBST for 1 h at RT. The samples were then washed 5 times for 5 min each with PBST and incubated with Hoechst 33258 at a concentration of 5 µg/mL in DPBS^+/+^ for 15 min at RT. After washing twice with DPBS^+/+^, cells were mounted with coverslips using fluorescence mounting medium (Dako, S3023) and stored at 4 °C until imaging with an inverted confocal microscope (TCS SP8, Leica Microsystems, Wetzlar, Germany).

### Flow cytometry of iPSC-derived CMs

For flow cytometry analysis of cTnT, iPSC-CMs at day 15 of differentiation were dissociated with papain and 5 × 10^6^ cells were fixed in 4% PFA for 15 min at RT. The samples were permeabilized with 0.25% Triton X-100 in DPBS^+/+^ for 15 min and blocked with 10% FBS in PBST for 1 h at RT. Afterwards, the cells were incubated with primary antibody for cTnT (Thermo Fischer Scientific, MA5-12960) or IgG isotype control in 1% FBS in PBST on a shaker overnight at 4 °C. After three times washing with PBST, fluorescent dye-conjugated anti-rabbit secondary antibody was incubated for 1 h at RT. The cells were measured on the Gallios flow cytometer (Beckman Coulter, Germany) and data were analyzed using Kaluza software version 1.2 (Beckman Coulter).

### RNA isolation, reverse transcription PCR (RT-PCR), and quantitative real-time PCR (qRT–PCR)

For qRT-PCR analysis, total RNA was isolated from iPSCs, iPSC-derived cardiac progenitor cells (d6) and iPSC-derived CMs (d22 and d60) using the Absolutely RNA Microprep kit (Agilent, 400805) or Nanoprep kit (Agilent, 400753), depending on the amount of starting material, following manufacturer’s instructions. Up to 1 μg of RNA was used to synthesize cDNA with the High-Capacity cDNA Reverse Transcription kit (Applied Biosystems, 4368814) with RNase inhibitor (Invitrogen, 10777-019) according to the manufacturer’s instructions. Gene expression was quantified by qRT-PCR using 1 μL cDNA and the Power SYBR Green PCR Master Mix (Applied Biosystems, 4368706). Then, the reaction was run on a 7500 Fast Real-Time PCR instrument (Applied Biosystems, Germany). The mRNA expression levels of PPCS were quantified relative to GAPDH. Following primers were used for PPCS (forward 5′-CCACTTTGGCGGACTATTTG-3′; reverse 5′-TTCAGGCATTTCAGAGACAGG-3′) and GAPDH (forward TCCTCTGACTTCAACAGCGA; reverse GGGTCTTACTCCTTGGAGGC) qRT-PCR.

### Treatment of cells with pantethine and 4’-P-pantetheine and measurements of cellular CoA

Pantethine (Sigma, #16702) and 4’-P-pantetheine (custom synthesized by Symeres, 97% by HPLC/MS, batch no. 30808-2) were added to cell type-specific cell culture media. Both compounds were used at 50, 150, and 500 µM without causing cell toxicity. Pantethine treatment was done in a culture medium with heat-inactivated serum. In case of fibroblasts, pantethine and 4’-P-pantetheine were added to cell culture media 2 h after seeding cells. Five days after the initial treatment, cells were lysed (Abcam, ab179835), spun down, and supernatants collected for CoA measure with a fluorescent-based kit following the manufacturer’s instruction (Abcam, ab138889). Finally, CoA values were normalized to total protein content (Bio-Rad, Bradford Assay, #500-0006).

In the case of fibroblasts, 800 cells/well were seeded on day 0 in 96-well plates. No medium change was performed during the 5 days treatment.

In the case of iPSCs, 15000 cells/well were seeded on day 0 on 96-well geltrex-coated plates in E8 medium with 10 μM Thiazovivin. Next day, the medium was removed and replaced by E8 supplemented with pantethine. Medium change was performed every day during the 5 days treatment.

For cardiac precursors, 25000 iPSCs/well were seeded in 96-well geltrex-coated plates in E8 medium with 10 μM Thiazovivin. Next day, medium was replaced by E8. When cells reached 95% confluency (day 4 after reseeding), cardiomyocyte differentiation was induced by changing to RPMI1640 containing B27 insulin minus (basal cardiac differentiation medium) supplemented with 1 µM CHIR-98014 and pantethine (d0). Next day, the medium was replaced with basal cardiac differentiation medium, supplemented with pantethine. On day 3, medium was replaced with basal cardiac differentiation medium supplemented with 2 µM Wnt-C59 and pantethine. CoA measurements were performed on day 6 of cardiac differentiation.

For CMs, d15 and d53 CMs were dissociated and seeded at the density of 50000 cells/well on day 0 in 96-well fibronectin-coated plates in EB20 medium (DMEM/F12 containing 20% heat-inactivated FBS, 1x MEM nonessential amino acids, 1x penicillin/streptomycin, 0.1 mM β-mercaptoethanol and 2 mM L-glutamine), Next day, medium was changed to EB2 (DMEM/F12 containing heat-inactivated 2% FBS, 1x MEM nonessential amino acids, 1x penicillin/streptomycin, 0.1 mM β-mercaptoethanol and 2 mM L-glutamine), pantethine was applied in EB2 medium two days after reseeding and medium change was performed every other day. CoA measurements were performed on day 5 after initial pantethine treatment.

### Statistical analyses

All statistical analyses were conducted using GraphPad Prism version 9.2.0 or 10.3.0 (GraphPad Software, San Diego, CA). For all experiments, at least two independent biological replicates were analyzed. Where applicable, experiments included multiple technical replicates. The exact number of biological replicates is provided in the figure legends. Bar or line graphs indicate the mean ± standard deviation (SD) or standard error of the mean (SEM), combining replicates from within and across experiments. Box plots display all data points and indicate the median and 25th and 75th percentiles, with whiskers extending to the min and max values. Statistical significance was determined using an independent samples *t*-test or analysis of variance (one-way or two-way ANOVA) for normally distributed data and Kruskal-Wallis test for non-normally distributed data. The statistical tests used for each dataset are specified in the figure legends. For Western blot analyses, representative images are shown, with quantification corresponding to the displayed blot. Each experiment was independently repeated multiple times to confirm the reproducibility and consistency of the results. The exact *p* values are indicated in the graphs or figure legends. A *p* value of < 0.05 was considered statistically significant.

### Reporting summary

Further information on research design is available in the Nature Portfolio Reporting Summary linked to this article.

## Results

### Patients with known and unreported pathogenic variants in PPCS present DCM accompanied by neurological and other extracardiac features

The study identified additional six patients compared to previously reported cases (Table S3), increasing the total number of identified PPCS DD cases to twelve (Table 1). The clinical and genetic conditions are detailed in the following case reports.

**Table 1:** Demographic, clinical and laboratory findings in PPCS DD patients

Literature	Current manuscript	Current manuscript	Current manuscript	Current manuscript	Current manuscript	Current manuscript	Iuso et al. 2018 and current manuscript	Iuso et al., 2018 and current manuscript	Iuso et al., 2018 and current manuscript	Iuso et al., 2018 and current manuscript	Iuso et al., 2018 al., and current manuscript	Lok et al., 2022
Individuals	F1:II	F2:II.1	F3:III.2	F3:III.3	F4:II	F5:IV.1	F6:IV.1	F6:IV.4	F6:IV.5	F6:IV.8	F7:II.2	
Consanguinity	No	Yes	No	No	Yes	Yes	Yes	Yes	Yes	Yes	No	No
Age of presentation	9 mths	Newborn	21 yrs	17 yrs	Antenatal	14 yrs	2 yrs	4 mths	3 yrs	23 mths	2 weeks	Newborn
Status	Alive, 11 yrs	Death, 3 mths, DCM, metab. acidosis	Alive	Alive	Death, 10 months, cardiac arrest	Alive, 19 yrs	Death, 21 yrs, SARS-CoV-2 complications	Alive, 14.5 yrs	Death, 3 yrs, DCM	Death, 23 mths, DCM	Death, 3 mths, DCM, multi organ failure	Death, 4 mths, DCM, multi organ failure
Therapeutic approach	Pantethine	n.a.	Metoprolol	Heart transplant	Supportive	Pantethine	Pantethine	Pantethine	n.a.	n.a.	n.a.	n.a.
Cardiac manifestation	Severe DCM	Severe DCM	LV non-compaction CM	Severe DCM	Septal hypertrophy	DCM	DCM	DCM	Severe DCM	Severe DCM	Severe DCM	Severe DCM
Extra cardiac manifestation	Hypotonia, rhabdomyolysis	n.d.	None	None	Rhabdomyolysis, liver dysfunction (hyperammonaemia, transaminitis, coagulopathy)	None	None	None	None	None	Severe hypotonia, dysmorphic features	Hypotonia, rhabdomyolysis,dysmorphic features
Serum CK	Elevated	Elevated	n.d.	n.d.	Elevated	n.d.	Normal	Normal	n.d.	n.d.	Elevated	Elevated
Serum lactate	Elevated	Elevated	n.d.	n.d.	Elevated	n.d.	Normal	Normal	Elevated	Elevated	Elevated	Normal
Hyperammonaemia	n.d.	n.d.	n.d.	n.d.	n.d.	n.d.	n.d.	n.d.	n.d.	n.d.	n.d.	n.d.
Acyl carnitine	Elevated	Elevated	Normal	Normal	Elevated	n.d.	Normal	Elevated	n.d.	Elevated	Elevated	n.d.
Brain MRI	Loss brain parenchyma, prominent sulci, ventricles	Normal	n.d.	n.d.	Non-specific changes suggesting oedema	n.d.	Normal	n.d.	n.d.	n.d.	Normal	Normal
Mutation in PPCS	c.[232 T > C]; [232 T > C] p.[Tyr78His];[Tyr78His]	c.[317 G > C]; [317 G > C] p.[Arg106Pro];[Arg106Pro]	c.[698 A > T]; [698 A > T] p.[Glu233Val];[Glu233Val]	c.[698 A > T]; [698 A > T] p.[Glu233Val];[Glu233Val]	c.[59 C > G];[59 C > G] p.[Ala20Gly];[Ala20Gly]	c.[698 A > T]; [698 A > T] p.[Glu233Val];[Glu233Val]	c.[698 A > T]; [698 A > T] p.[Glu233Val];[Glu233Val]	c.[698 A > T]; [698 A > T] p.[Glu233Val];[Glu233Val]	c.[698 A > T]; [698 A > T] p.[Glu233Val];[Glu233Val]	c.[698 A > T]; [698 A > T] p.[Glu233Val];[Glu233Val]	c.[538 G > C]; [320_334del] p.[Ala180Pro]; [Pro107_Ala111del]	c.[613-3 C > G]; [320_334del] p.[?]; [Pro107_Ala111del]

*n.d*. Not determined.

Patient F1:II is currently 12 years old. He was born at term with normal growth parameters following an unremarkable pregnancy and neonatal course. He initially presented at 9 months of age after 1 week of upper respiratory symptoms and 1 day of vomiting and diarrhea. In the emergency department, he became unresponsive and was found to be hypoglycemic with a blood glucose level of 1.3 mol/L. He became bradycardic and hypotensive and required cardiac resuscitation. He was admitted to the pediatric intensive care unit (PICU) for supportive care. Echocardiogram demonstrated normal cardiac size and function.

Initial metabolic evaluation revealed elevations in multiple long-chain fatty acyl-CoA species suggestive of a fatty acid oxidation defect. Disproportionate elevations were noted in C14:1 (4.78 mmol/L, normal 0-0.3) and C14:2 (3.07 mmol/L, normal 0-0.15) suggestive of very long-chain acyl-CoA dehydrogenase deficiency (VLCADD). Urine organic acid analysis revealed a pattern of lactic aciduria and modest ketonuria with inappropriate excretion of medium to longer chain dicarboxylic acids of chain lengths C6-C14. Unsaturated dicarboxylic acids were present, suggesting impaired fatty acid oxidation. Creatine kinase (CK) level was normal. Dietary therapy for VLCADD was initiated, and he recovered and was discharged home. Follow-up molecular testing, including ACADVL sequencing, a next-generation glycogen storage disease panel, and a next-generation mitochondrial disease panel (which included several genes for fatty acid oxidation disorders), was unrevealing. He was found to harbor a homozygous variant of uncertain significance (VUS) in MCCC1 (p.Thr407Asn) but had no biochemical evidence of 3-methylcrotonyl-CoA carboxylase (3MCC) deficiency. As 3MCC deficiency is not associated with cardiomyopathy and thought to be a relatively benign biochemical anomaly, this was not felt to be the cause of his symptoms.

He had no further health concerns until 34 months of age when he again developed upper respiratory symptoms and poor feeding. Blood glucose levels were normal; however, cardiomegaly was noted on the chest radiograph. Echocardiogram demonstrated severe dilation and global dysfunction of the left ventricle (LV). His LV internal diameter at end diastole measured at a z-score of 4.7 and in systole at z-score of 9.75. Ejection fraction was 15%, with a 9% shortening fraction. He was started on inotropic support and ultimately required 11 days of extracorporeal membrane oxygenation (ECMO). Myocardial biopsy was not consistent with myocarditis. Metabolic testing at this time did not show signs of impaired fatty acid oxidation. Endomyocardial biopsy demonstrated mild focal interstitial fibrosis and many myocytes showing nucleomegaly. There was no increase in inflammatory cells, myocyte damage, or necrosis. Electron microscopy demonstrated a normal complement of mitochondria with preserved cristae.

Genetic evaluation was extended to include a SNP microarray which showed no copy number variants but did contain multiple regions of homozygosity. Clinical exome sequencing and mitochondrial DNA (mtDNA) sequencing were unrevealing. He improved clinically and was discharged after approximately 6 weeks. Over the following year, he had normalization of cardiac function and size.

He had a subsequent episode of acute heart failure at 4.5 years of age in the setting of an upper respiratory tract infection requiring 12 days of ECMO support. He was not hypoglycemic at this time. He was also noted to have developed a prolonged QT interval on electrocardiogram (ECG). He subsequently developed muscle pain and weakness of unclear etiology, with a normal CK value at the time. Brain MRI demonstrated global parenchymal volume loss with prominent sulci and ventricles. There were also scattered nonspecific foci of T2 prolongation in the subcortical and deep white matter, suspicious for past insults given his cardiac history. Skeletal muscle ultrasound was notable for fasciculations and heterogeneous appearance. Exome reanalysis demonstrated a VUS in the gene OGN encoding osteoglycin, a protein which induces ectopic bone formation in conjunction with transforming growth beta factor, therefore thought not to be associated with his disease. He had slow improvement in his cardiac function over the subsequent year. However, he developed episodes of rhabdomyolysis requiring hospital admission during intercurrent illness or periods of poor feeding with a peak lifetime CK level of 26,000 U/L (normal 75-230).

At age 6 years, he had an episode of worsening cardiac function in the setting of a viral illness. His LV ejection fraction declined from 58% to approximately 30%. He was started on intravenous milrinone which he required for several months. Given worsening skeletal muscle weakness, he also underwent skeletal muscle biopsy, which showed findings suggestive of a neurogenic process, with no findings suggestive of a mitochondrial disease. At age 7, he again had an episode of worsening cardiac function, requiring 5 days of milrinone therapy. His cardiac function normalized again after this episode.

**Fig. 1:**
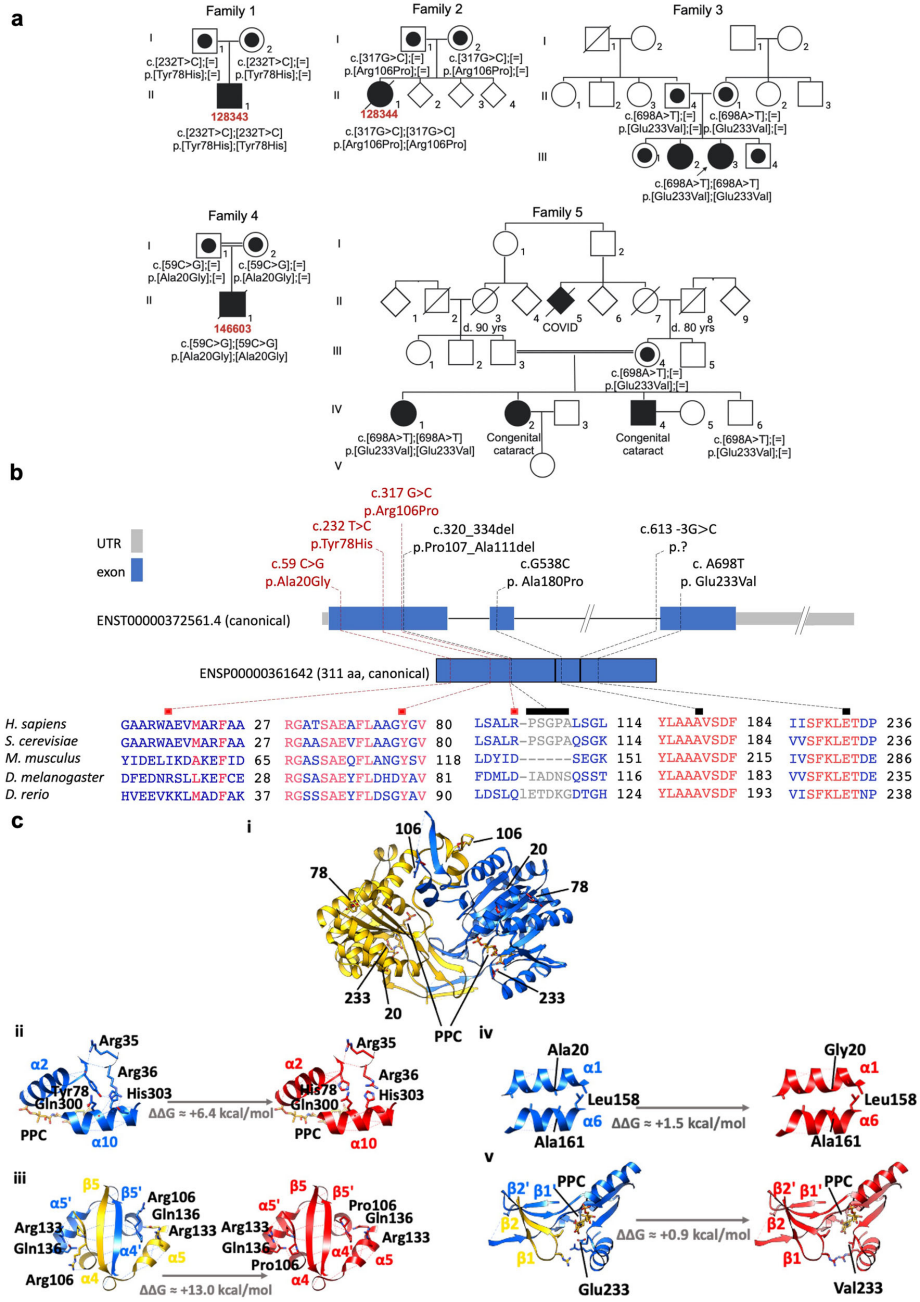
Newly identified variants occur in exon 1 of PPCS and are predicted pathogenic. **a** Pedigrees of the five families with mutations in PPCS. Affected individuals are indicated with closed symbols, healthy family members with open symbols. Carriers are denoted by a dot in the center of the circle or square symbol. Fibroblast identification numbers are in red. **b** Localization of the newly identified (red) and already reported (black) pathogenic variants in PPCS at the gene and canonical protein level, with a zoom in the conservation of amino acid residues affected by mutations. Coloring in the sequence alignment represents the identity of amino acid residues (COBALT alignment tool). Scale gene: 100 bp = 1 cm; scale proteins: 1 cm = 33 amino acids. **c** Effect of Tyr78His, Arg106Pro, Ala20Gly and Glu233Val on the 3D structure of human PPCS. (i) Ribbon representation of the human PPCS dimer 3D structure (PDB code 1P9O) and side chain stick representation of Tyr78, Arg106, Ala20 and Glu233 in WT, and of His78, Pro106, Gly20 and Val233 in the thereof homology models produced with FoldX. Chain A and B of the WT protein are colored in yellow and blue, respectively, whilst residues from the mutants are colored in red. The enzymatic product analog phosphopantothenoylcystine (PPC) from the crystal structure of S. cerevisiae PPCS is shown in orange. Zoom in of the WT (left) and FoldX homology models (right) in the mutation region of (ii) Tyr78His, (iii) Arg106Pro, (iv) Ala20Gly and (v) Glu233Val highlighting residues and hydrogen bonds (dashed lines) relevant for stabilization. The ΔΔG values in kcal/mol between the WT and mutant proteins were calculated using FoldX, and account for the change in free energy for the apoprotein upon mutation, with positive values suggesting destabilization of the 3D structure and pathogenicity. The structure models in the figure were produced using UCSF Chimera.

Exome reanalysis at this time was notable for a homozygous variant in PPCS c.232 T > C, p.Tyr78His (Family 1, Fig. 1a, b, Table 1). Clinical RNA sequencing of his skeletal muscle tissue confirmed the presence of this variant in expressed transcripts and an overall reduction in PPCS expression levels compared to controls. Given this finding, 450 mg (17 mg/kg/day) of oral pantethine was initiated at the age of 8 years. The dose was well tolerated and subsequently increased to 900 mg/day at 9 years. Since initiating pantethine, he has not had any further episodes of rhabdomyolysis. In addition, he reports improvement in appetite and energy level. However, he continues to have muscle weakness requiring a wheelchair to assist with ambulation. His most recent skeletal muscle ultrasound was consistent with a neuromuscular process with no convincing fasciculation. Brain MRI demonstrated no evidence of iron accumulation. At the last clinical cardiac evaluation, LV size was normal with low-normal function. Interestingly, on metabolic testing, he had never shown any evidence of 3MCC deficiency; however, after starting pantethine he began to excrete 3-methylcrotonylglycine in his urine.

Patient F2:II.1 died at 3 months of age. She was born at 38 + 5 weeks of gestation. APGAR scores were 7 and 8. She required continuous positive airway pressure (CPAP) support for respiratory distress after delivery and was admitted to the neonatal intensive care unit (NICU). Polysomnogram demonstrated central apneas, which required treatment with caffeine. A gastrostomy tube was placed as she was noted to aspirate with oral feeds. Echocardiogram obtained during her NICU stay showed normal biventricular size and function, a small patent foramen ovale with left to right shunt, a small patent ductus arteriosus with left to right shunt, and a trivial apical muscular ventricular septal defect. She was noted to have staring episodes with limb jerking. Electroencephalogram and brain MRI were unremarkable. She was discharged home at 49 days of life.

One week after discharge from the NICU, she presented to the emergency department with worsening upper respiratory congestion and shortness of breath. She was noted to have an apneic episode in the emergency department. She was admitted to the PICU for respiratory support and was found to be cytomegalovirus positive. She required increasing respiratory support and was ultimately intubated. She developed hypothermia and metabolic acidosis, requiring sodium bicarbonate supplementation. Echocardiogram demonstrated mild LV dilation with an ejection fraction of 51%, which worsened to 28% 24 h later. She required escalating ionotropic support. Cardiac MRI showed hyperemia and capillary leak consistent with possible myocarditis. The LV was dilated with a LV internal diameter at end diastole measured at a z-score of 3.4 and in systole at z-score of 5.2. Her hospital course was further complicated by Pseudomonas pneumonia, candida fungemia, and a Klebsiella urinary tract infection. She died approximately one month after admission, when she developed worsening acidosis and malignant tachyarrhythmias refractory to medical management.

Metabolic testing was unremarkable, including urine organic acid, plasma amino acid, serum lactate/pyruvate, N-glycan, and serum acylcarnitine analysis. Serum and urine total carnitine levels were elevated, reflecting carnitine supplementation administered to support her cardiac function. CK levels were elevated with a peak recorded value of > 3200 U/L (normal 60-305 U/L). SNP microarray demonstrated a 437 kb duplication of 1p31.1, arr[hg19] chr1:72,503,891-72,941,284 ×3. This duplication contains exon1 of NEGR1, which is not known to be associated with human disease. In addition, she was noted to have multiple areas of homozygosity on microarray. Clinical exome sequencing revealed the homozygous variant c.317 G > C, p.Arg106Pro in PPCS (Family 2, Fig. 1a, b, Table 1). She also carried a single maternally-inherited VUS (c.1255 G > A) in GAA encoding acid alpha glucosidase (GAA) but had normal GAA enzyme testing. In addition, she had a maternally-inherited VUS in LMNA (c.1685 T > A); however, her mother had no cardiac concerns, and LMNA-associated cardiomyopathy is typically an adult-onset diagnosis.

Cascade testing of the PPCS variant revealed that each of her parents carried one copy of the PPCS variant. Her three full siblings had normal cardiac evaluations and none was homozygous for the PPCS variant.

Patient F3:III.3 is a 16-year 7-month female with no known past medical history. She presented following an out-of-hospital cardiac arrest. In the 2 weeks preceding her event, she noted multiple episodes of near syncope. She had participated in field hockey practice the morning of her event without symptoms. While at home visiting with family, she collapsed, and bystander cardiopulmonary resuscitation (CPR) was performed. Upon arrival of emergency medical services, she was found to be in ventricular fibrillation, for which she was successfully defibrillated. Coronary anomalies were ruled out by prior computed tomography angiography and echo. No other clinical or imaging evidence to suggest obstructive coronary artery disease. Over the next 3 days, she had recurrent ventricular arrhythmias despite multiple antiarrhythmic medications and cardiac pacing. Echocardiogram demonstrated mild dilation of the LV and moderate to severely depressed LV systolic function (echo prior to ECMO with LV FS 23, LV EF A4C 46%, LV EF A2C 43%). Cardiac MRI did not demonstrate significant fibrosis or inflammation. Endomyocardial biopsy did not demonstrate acute myocarditis but did reveal histopathologic features most suggestive of underlying cardiomyopathy (abnormal disorganized myocytes with nuclear changes). Due to recurrent ventricular arrhythmias requiring defibrillation, she was placed on veno-arterial ECMO. Critical trio whole exome sequencing with mtDNA analysis was performed and identified a homozygous pathogenic variant in the PPCS gene, c.698 A > T, p.Glu233Val (Family 3, Fig. 1a, b, Table 1). Parents are heterozygous carriers of the PPCS variant. Acylcarnitine profile was normal.

After multidisciplinary discussions, the patient was transferred from the Children’s Hospital to the local adult medical center for placement of a total artificial heart (TAH) as a bridge to transplant. The post-op course was complicated by acute kidney injury requiring hemodialysis (HD), inability to close her chest, right femoral thromboembolism requiring embolectomy, fasciotomy, and ultimately right lower extremity below-the-knee amputation. Fifteen days after TAH placement, a suitable donor heart was identified, and the patient underwent successful heart transplantation. The post-transplant course was complicated by ongoing renal failure requiring HD, gastrointestinal bleeding, pneumonia, and malnutrition. Despite the multiple post-transplant complications, the patient eventually recovered and was discharged to acute rehabilitation on post-transplant day 52. She was completely liberated from HD. At nearly one-year post-transplant, she has had no rejection or graft dysfunction. She has returned to school and is playing field hockey again.

Patient F3:III.2 is a 21-year-old female with no significant past medical history who presented to clinic for cardiac screening based on her family history of cardiomyopathy (sister of patient F3:III.3). She reported an elevated heart rate of around 190 bpm with exercise and occasional palpitations but was otherwise asymptomatic from a cardiac perspective. ECG and echocardiogram were performed as part of her evaluation. ECG was significant for sinus tachycardia, low voltage QRS, and T-wave inversion in inferolateral leads. Echocardiogram demonstrated mildly diminished LV systolic function and increased LV trabeculations. Based on her sister’s history, she was admitted for monitoring and additional investigations including cardiac MRI (LV EF of 45%, noncompacted to compacted ratio of 2.7:1 in the inferolateral apical region), stress ECG, implantable loop recorder (no concerning arrhythmias to date), and acylcarnitine profile (values in the normal range). Single gene sequencing of PPCS identified homozygosity for the familial pathogenic PPCS variant, NM_024664.3:c.698 A > T, p.Glu233Val (Family 3, Fig. 1a, b, Table 1). She meets criteria for LV non-compaction cardiomyopathy, is managed with metoprolol, and is doing well.

Patient F4:II was the third child of consanguineous parents, born at 38 + 1 weeks’ gestation after a pregnancy complicated by gestational diabetes treated with metformin and maternal hypothyroidism and lupus treated with azathioprine and hydroxychloroquine. Antenatal ultrasound revealed suspicion of a ventricular septal defect, but postnatal ultrasound demonstrated cardiac muscle septal hypertrophy. She developed respiratory distress soon after birth and required CPAP in NICU. Chest radiograph suggested interstitial lung disease, and she had a persistent oxygen requirement. She had recurrent episodes of suspected sepsis in early infancy, associated with metabolic acidosis and elevated blood lactate levels (max 5 mmol/L, reference < 2), but infection markers and cultures were all normal. At 3 months she had an episode of rhabdomyolysis, with CK 71,000, associated with mild acidosis, high lactate and electrolyte disturbance secondary to diabetes insipidus. MRI brain revealed non-specific changes suggestive of oedema, but no focal lesions. Subsequently at 3.5 months she developed severe hyperammonaemia (max 1351 μmol/L, reference range < 40), for which she was treated with carglumic acid, continuous veno-venous haemofiltration and a protein restricted diet. Plasma amino acids revealed strongly elevated lysine (964 μmol/L, reference 100–300) and alanine (903 μmol/L, reference 150–450), the latter in keeping with lactic acidosis. Fibroblast growth factor 21 level, taken to investigate a suspected mitochondrial disorder, was elevated at 1881 pg/ml (reference 515). Acylcarnitine when unwell was suggestive of VLCADD, with strongly raised tetradecenoylcarnitine (C14:1) and tetradecanoylcarnitine (C14:2), moderately raised dodecanoylcarnitine (C12) and hexadecanoyl (palmitoyl)carnitine (C16). When well, the acylcarnitine profile was completely normal. Urine organic acids revealed strongly raised lactate and pyruvate with moderately raised 2-hydroxybutyrate and mildly raised 2-oxo-isocaproate (6 µmol/mmol creatinine), very strongly raised 3-hydroxybutyrate with strongly raised acetoacetate, strongly raised adipate, suberate, sebacate and 3-hydroxysebacate with moderately raised decendioate, 2-hydroxysebacate and 5-hydroxyhexanoate and mildly raised 3-hydroxyoctanoate, and mildly raised methylcitrate, 3-hydroxypropionate and 2-methyl-3-hydroxybutyrate and moderately raised 4-hydroxyphenyllactate (61 µmol/mmol creatinine) and 4-hydroxyphenylpyruvate with mildly raised vanillyl-lactate.

At 9.5 months she presented with persistent cough and retching, associated with mild acidosis, mildly elevated blood lactate (3.7 mmol/L), hyperammonaemia (max 117 μmol/L), coagulopathy and transaminitis (ALT max 450 μmol/L, reference < 33). She died at 10 months following two cardiac arrests from which she could not be resuscitated. Mitochondrial genome sequencing and rearrangement screen were normal. Trio exome sequencing revealed a homozygous missense variant NM_024664.4:c.59 C > G, p.Ala20Gly in PPCS (Family 4, Fig. 1a, b, Table 1).

Patient F5:IV.1 is a 19-year-old female; she is the youngest of four siblings, born to related parents. Two of her siblings had juvenile cataracts; otherwise the family history was unremarkable. Her medical history is remarkable for anxiety disorder as a child and a syncope at the age of 14, when investigations revealed a prolonged QT interval up to 560 ms which later normalized. Stress 24 h ECG and echocardiogram were normal. She was lost to follow-up until her most recent admission, when she was found semi-conscious with sudden-onset shortness of breath. She became pulseless, and CPR was initiated. The first ECG on the external defibrillator demonstrated ventricular fibrillation, which was successfully cardioverted to sinus rhythm. The patient was transferred to the Cardiac Intensive Care Unit (CICU). During her stay in CICU she was sedated, intubated, and treated with vasopressors. Chest CT demonstrated bilateral aspiration with no evidence of pulmonary embolism. She was supplemented for mild hypokalemia and hypomagnesemia. ECG on admission demonstrated sinus rhythm, nonspecific T wave inversion with no evidence of ischemia and prolonged QT up to 624 ms. ECG monitoring over 72 h did not demonstrate any arrhythmia. High-precordial leads ECG excluded Brugada syndrome. Toxic screen was negative. Echocardiography showed moderate to severe LV dysfunction, without significant valvular abnormalities. Cardiac MRI demonstrated dilated LV with severe biventricular dysfunction with LV ejection fraction of 34%, right ventricle ejection fraction of 37% and no significant late gadolinium enhancement.

The patient was successfully extubated without any neurological deficit. She went home after implantable cardioverter-defibrillator (ICD) insertion, on beta-blockers, ACE-inhibitors, and 600 mg/day pantethine. At a three-month follow-up the patient felt well, ICD interrogation did not demonstrate any arrhythmia and echocardiography revealed fully recovered LV function. A year later, the ICD recorded a short run of asymptomatic ventricular tachycardia that resolved spontaneously. Repeat ECG demonstrated QT within normal limits, and echocardiography demonstrated normal LV function.

Whole Exome Sequencing (WES) revealed homozygosity for a PPCS variant c.698 A > T; p.Glu233Val (Family 5, Fig. 1a, b, Table 1).

Additionally, two other pertinent genetic alterations emerged: (i) KCNH2: c.3457 C > T; p.His1153Tyr. Pathogenic KCNH2 gene variants associate with Long QT syndrome type 2. The variant c.3457 C > T; p.His1153Tyr, has been reported in a patient with Long QT syndrome (PMID: 16414944) and in a sudden death case (PMID: 26164358), and holds uncertain clinical significance (ClinVar Variation ID: 67497), and (ii) FYCO1:c.832_833del; p.Arg278GlyfsTer19. Pathogenic FYCO1 variants may induce nonsyndromic autosomal recessive congenital cataracts. This might account for the cataracts observed in two of the patient’s siblings.

The patient’s mother and one of her brothers underwent comprehensive clinical evaluations. The mother (III.4) was found to have mildly prolonged QT with normal exercise test and normal LV function. ECG, exercise test and echocardiography were normal in the brother (IV.6).

Genetic testing revealed that both the mother and brother are heterozygous for the familial PPCS variant, in keeping with the absence of cardiomyopathy symptoms in both individuals.

Notably, the variant in KCNH2 did not exhibit the expected segregation pattern. The mother, despite not being a carrier, clinically manifested prolonged QT, while the son carried the variant without clinical signs. Finally, the mother was heterozygous for the FYCO1 change, and the son did not carry this gene alteration.

### Pantethine-treated patients exhibit encouraging benefits

To date, four patients with PPCS DD have received pantethine supplementation.

F1.II has not had any further episodes of rhabdomyolysis since he initiated pantethine; he reported improvement in appetite and energy level, although he continues to have muscle weakness and requires a wheelchair to assist with ambulation. His most recent skeletal muscle ultrasound was consistent with a neuromuscular process with no convincing fasciculation. At the last clinical cardiac evaluation, his LV size was normal with low-normal function (Fig. S2). Laboratory parameters, including creatine kinase (CK) and B-type natriuretic peptide (BNP), decreased and stabilized within normal ranges, with CK levels below 365 U/L and BNP levels below 100 pg/mL.

F5:IV.1 has not had any further episodes of arrhythmia and echocardiography revealed fully recovered LV function since she commenced pantethine supplementation (Fig. S2).

The longest treatment duration, started in 2018, was for individuals F6:IV.1 and F6:IV.4 from Family 6, initially described in ref. ^2^ (Fig. S3). In both patients, we observed a mild improvement in exertional dyspnea and an increase in the ejection fraction^2^. In April 2021, both affected siblings were found to be SARS-CoV-2 positive and showed respiratory symptoms necessitating hospitalization and ICU admission. The younger sibling, at 14.5 years of age, was discharged home after nearly two weeks. Unfortunately, his 21 year-old brother had a much more severe course, required ECMO support, and sadly succumbed after a month-long hospitalization. Of note, the cardiac function of both siblings was stable during the past years of follow-up, including throughout the COVID-19 infection.

### Prediction analysis of protein structure stability of the variants supports pathogenicity

PPCS forms a dimer, and the oligomerization domain is critical for its biological function^20,21^. FoldX Suite^22^ was used to evaluate the potential effect of the newly identified missense variants on the 3D structure and function of PPCS. This program is one of the few programs that can deal with multimer structures and performs energy minimization on the structures. Therefore, it is currently one of the best programs to evaluate the effect of missense variants on the three dimensional (3D) protein structures^31^. The 3D structure of the wild-type (WT) PPCS (PDB: 1P9O) was first minimized and then used to produce the 3D models of the mutant proteins (Fig. 1c,i). The effect of variants on the stability of the proteins and their potential pathogenicity was then computed by comparing the Gibbs free energies (ΔΔG) of the mutants with that of the WT protein^22,31^ (Fig. 1c,ii-v). All four variants (Glu233Val, Ala20Gly, Tyr78His, and Arg106Pro) are predicted to cause positive ΔΔG values between + 0.9 (p.Glu233Val) and + 13.0 kcal/mol (p.Arg106Pro) and are therefore expected to destabilize the protein and be pathogenic. The ΔΔG value of + 13.0 kcal/mol for p.Arg106Pro most probably underestimates the effect of the variant because the WT model has the residues 110-112 and 108-112 missing in chains A and B, respectively, and the residues around the variant, including Arg106, have high B-factors, i.e., the position of the side chain does not have high accuracy due, for example, to dynamics.

The mutation Tyr78His does not seem to cause significant alterations in the conformation of the protein nor clashes (Fig. 1c,ii), but because the sidechain of Tyr78 maintains a network of hydrogen bonds stabilizing the α1-β1 loop interactions with helices α2 and α10, mutation to His78 will lead to a disruption of the hydrogen bond network, stability, and dynamics. Since these changes are in the close vicinity of the catalytic site, it is expected that they will cause changes in the catalytic activity of PPCS.

Mutation Arg106Pro affects the dimerization region between helices α4 and α5, which in humans includes a non-conserved sequence that forms a β-sheet between the two monomers (Fig. 1c,iii). Variant p.Arg106Pro will most probably disturb the β-sheets involved in the dimerization, especially because residue 107 is also a proline. The effect of the variant cannot be adequately evaluated because the region of the variant has high B-factors or does not have electron density. This hampers proper modeling of the variant and evaluation of the destabilizing effect. Nevertheless, it is expected that the p.Arg106Pro variant will increase the dynamics in the region and destabilize the β-sheet, which is essential for maintaining the dimer needed for catalytic activity. In fact, FoldX predicts a ΔΔG + 13.0 kcal/mol for this the variant, though the destabilizing effect is probably underestimated.

The mutation Ala20Gly seems to cause a minor effect on the stability of the protein of only approximately + 1.5 kcal/mol and without altering the secondary structure or causing clashes (Fig. 1c,iv). However, because helices α1 and α6 are stabilized by hydrophobic contacts between mainly side chain of alanines, it is possible that this change alters the stability of the helices or even their dynamics. This could have an effect on the catalytic activity of the mutant protein.

The mutation Glu233Val has the smallest predicted effect on the apoprotein stability (0.9 kcal/mol), as it does not seem to cause changes in secondary structure, hydrogen bonding capability or packing of the protein (Fig. 1c,v). However, Glu233 is in the active site of the enzyme and close to a dimerization interface of the enzyme and should be important for the stabilization of the ATP substrate as it has been suggested by work on the homologue E. coli enzyme^32^. Therefore, it is expected to have a significant effect on the activity of the enzyme.

### Variants in PPCS affect protein stability in patient-derived fibroblasts and lead to decrease in cellular levels of CoA, ameliorated by supplementation of CoA intermediates

**Fig. 2:**
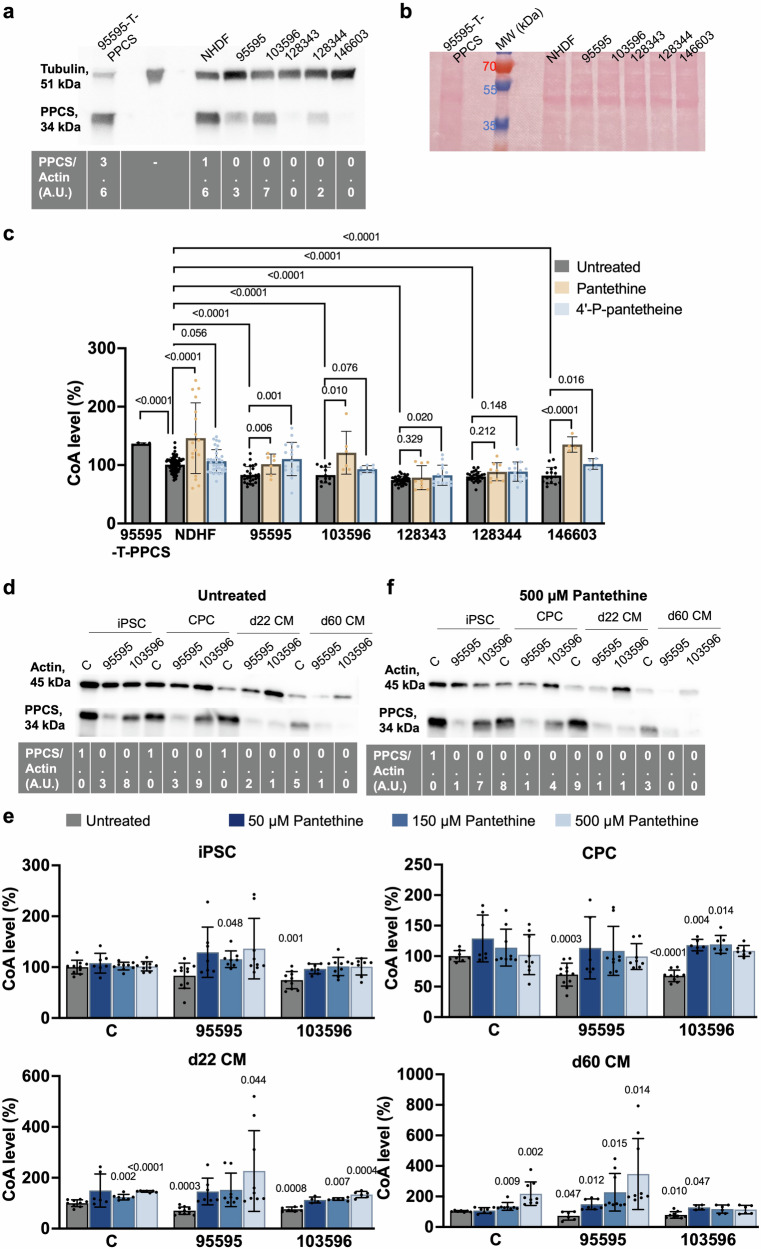
Pathogenic variants in PPCS lead to reduced intracellular CoA restored through the supplementation of pantethine and 4’-P-pantetheine. **a** Representative western blot analysis of fibroblasts 128343, 128344 and 146603 from newly identified patients. Fibroblasts 95595 and 103596 from previously reported patients were included as positive controls, while fibroblasts 95595 overexpressing WT PPCS (95595-T-PPCS) and NHDF from a healthy subject, were used as healthy control samples. 30 µg of total protein lysates were loaded on a 16% Tris-Glycine gel. Tubulin was used as a loading control. At the bottom of the western blot is reported the densitometric analysis of PPCS normalized to the housekeeping protein tubulin in arbitrary units (A.U.). The densitometric analysis is relative to the shown experiment. Uncrop western blot in Fig.S10. **b** Ponceau staining of the membrane shown in panel (a). Uncrop Ponceau staining in Fig. S11. **c** Intracellular levels of CoA in fibroblasts from PPCS DD patients 95595, 103596, 128343, 128344 and 146603. Fibroblasts from patient 95595 overexpressing WT PPCS (95595-T-PPCS) and NHDF were used as healthy control subjects. CoA levels in untreated NHDF were set to 100 and levels in the other fibroblast lines expressed as percent (%) of NHDF values. Measurements were performed in standard growth conditions (untreated) and in presence of 500 µM pantethine and 500 µM 4’-P-pantetheine. Results are presented as mean ± SD. Individual sample sizes are indicated for each condition (untreated: 95595-T-PPCS *n* = 4, NDHF *n* = 79, 103596 *n* = 13, 128343 *n* = 34, 128344 *n* = 26, 146603 *n* = 16; pantethine: NDHF *n* = 17, 95595 *n* = 7, 103596 *n* = 6, 128343 *n* = 9, 128344 *n* = 9, 146603 *n* = 4; 4’-P-pantetheine: NDHF *n* = 34, 95595 *n* = 17, 103596 *n* = 7, 128343 *n* = 14, 128344 *n* = 14, 146603 *n* = 4). Statistical significance relative to the untreated control was determined using an independent samples t-test, with exact *p*-values reported. **d**, **e** Western blot analysis of iPSCs, CPCs, d22 CMs and d60 CMs in standard growth condition (d, untreated) and after treatment with pantethine (e, 500 µM pantethine) from a healthy control subject (C) and PPCS DD patients (95595,103596). 5 µg of proteins were loaded on an 8–16% Tris-Glycine gels. Actin was used as a loading control. At the bottom of western blot is reported the densitometric analysis relative to the shown western blots. Uncrop western blot in Fig. S12. **f** Intracellular levels of CoA in iPSC, CPC, d22 CM and d60 CM from a healthy control subject (C) and PPCS DD patients (95595,103596). For each cell type, the levels of CoA levels in the untreated control were set to 100 and levels in the other cell lines express as percent (%) of control value. Measurements were performed in standard growth conditions (untreated) and in presence of 50, 150 and 500 µM pantethine. Results are presented as mean ± SD. Individual sample sizes are indicated for each condition (iPSC untreated: C *n* = 10, 95595 *n* = 11, 103596 *n* = 10; iPSC 50 µM pantethine: C *n* = 7, 95595 *n* = 7, 103596 *n* = 7; iPSC 150 µM pantethine: C *n* = 9, 95595 *n* = 8, 103596 *n* = 9; iPSC 500 µM pantethine C *n* = 9, 95595 *n* = 9, 103596 *n* = 8; CPC untreated: C *n* = 8, 95595 *n* = 11, 103596 *n* = 9; CPC 50 µM pantethine: C *n* = 7, 95595 n = 6, 103596 n = 6; CPC 150 µM pantethine: C n = 9, 95595 n = 9, 103596 n = 7; CPC 500 µM pantethine C *n* = 9, 95595 *n* = 8, 103596 *n* = 7; d22 CM untreated: C *n* = 9, 95595 *n* = 10, 103596 *n* = 7; d22 CM 50 µM pantethine: C *n* = 6, 95595 *n* = 7, 103596 *n* = 4; d22 CM 150 µM pantethine: C n = 7, 95595 n = 8, 103596 n = 6; d22 CM 500 µM pantethine C *n* = 7, 95595 *n* = 9, 103596 *n* = 6; d60 CM untreated: C *n* = 7, 95595 *n* = 6, 103596 *n* = 9; d60 CM 50 µM pantethine: C *n* = 7, 95595 *n* = 7, 103596 *n* = 4; d60 CM 150 µM pantethine: C *n* = 8, 95595 *n* = 9, 103596 *n* = 6; d60 CM 500 µM pantethine C *n* = 9, 95595 *n* = 9, 103596 *n* = 6). Statistical significance relative to the control was determined using an independent samples t-test, with exact *p*-values reported. Uncrop western blot in Fig. S13.

Since functional complementation analysis in yeast was inconclusive for determining the pathogenicity of the newly identified variants (Supplementary Methods, Supplementary Results, Supplementary References; Fig. S4), we performed immunoblotting to assess PPCS protein levels and stability. Protein extracts were prepared from fibroblasts of affected individuals carrying the homozygous variants Tyr78His (128343), Arg106Pro (128344), and Ala20Gly (146603), corresponding to families 1, 2, and 5. For comparison, we included fibroblasts from an unrelated healthy control (NHDF), a previously reported patient stably overexpressing wild-type PPCS (95595-T-PPCS) as negative controls, and two previously reported patients as positive controls: one with compound heterozygous variants (Ala180Pro and Pro107_Ala111del in 95595), and another with a homozygous Glu233Val variant (103596)^2^ (Fig. S3). Under denaturing conditions, the monoclonal PPCS antibody, directed against the C-terminal region of PPCS, detected in both positive control lines (95595-T-PPCS and NHDF, Fig. 2a) intense PPCS signals (normalized to the housekeeping protein tubulin) at the expected 34 kDa size, and weaker signals in three affected individuals (95595, 103596 and 128344, Fig. 2a). No signal or a signal at the antibody detection limit were obtained in patients 128343 and 146603 (Fig. 2a) suggesting a stronger destabilization effects of the variants Tyr78His and Ala20Gly on PPCS compared to Arg106Pro, and the previously reported Ala180Pro, Pro107_Ala111del and Glu233Val (Fig. 2a). A ponceau staining was performed to verify that comparable protein amounts were loaded for each sample (Fig. 2b).

Consistent with the decreased PPCS amount, intracellular CoA levels were also reduced in fibroblasts of the newly identified patients (128343, 128344, and 146603) compared to control lines (NHDF, 95595-T-PPCS), and in line with the previously reported patients^2,3^, two of which (95595 and 103596, Fig. S3) were included in our assay as positive controls (Fig. 2c, Untreated).

As pantethine was used on a compassionate basis in the treatment of PPCS DD patients^2^ but its efficacy in elevating CoA in PPCS deficient cell lines was never proved, we treated patients fibroblasts 95595 and 103596 with different concentrations of pantethine for five days and measured CoA at the end of the treatment. In parallel, we evaluated the efficacy of 4’-P-pantetheine, another CoA intermediate, predicted to be more stable than pantethine in the blood and therefore expected to rescue CoA levels more efficiently than pantethine. The levels of CoA normalized to levels of the untreated control (NHDF) in both patient cell lines (95595, 103596) treated with 50 µM pantethine, and increased significantly in the control. By using 150 µM and 500 µM pantethine, there was an increase of CoA levels in both patient lines, compared to their values before the treatment, with a significant increase above the levels of the untreated control in NHDF and 103596 (Fig. S5).

4’-P-pantetheine instead, led to an increase of intracellular CoA levels in the patients’ cell lines only at 500 µM. However, the increase remained below the untreated control threshold in both patient lines (Fig. S5). No cytotoxic effects were exerted by both compounds at any of the concentrations tested.

Because both compounds induced the greatest increase in CoA level in both patient lines when used at 500 µM, we supplemented patients’ fibroblasts with 500 µM of pantethine or 500 µM 4’-P-pantetheine. Both compounds induced an increase in CoA levels in all cell lines, although not all patient lines reached the average CoA level of untreated healthy control cells (Fig. 2c). The increase of CoA induced by both compounds was smaller than that observed in a genetically corrected patient cell line overexpressing the wild-type PPCS gene (95595-T-PPCS) (Fig. 2c).

### PPCS variants affect protein stability and lead to reduced levels of CoA in patient-derived iPSCs, cardiac progenitor cells, and cardiomyocytes

To investigate the molecular mechanisms of the cardiomyopathy associated with PPCS deficiency, we induced the cardiac differentiation of iPSCs from patients 95595 and 103596, described in ref. ^33^, alongside healthy control iPSCs (Fig. S6a). qRT-PCR analysis in iPSCs and iPSC-derived cardiac progenitor cells (CPCs) and cardiomyocytes (CMs) at different maturation stages (22 days-old, d22 CMs and 60 days-old, d60 CMs) showed similar PPCS transcript levels at all developmental stages, with no significant differences between patient and control cells (Fig. S6a, b). By contrast, western blotting demonstrated markedly reduced PPCS protein levels in patient populations at all stages compared to controls, suggesting impaired protein stability (Fig. 2d). The subcellular localization pattern of PPCS remained unchanged, however, with nuclear and cytosolic immunofluorescence signal detected in all iPSC-derived samples (Fig. S6c). Dual localization of PPCS was independently confirmed by immunocytochemistry analyses performed in HeLa and fibroblast cells transfected with GFP-tagged canonical PPCS (Fig. S6d).

We also detected a decrease in CoA levels in both patients’ iPSCs, although the decrease reached statistical significance only in patient 95595 (Fig. 2e). The defect became significant and more pronounced during cardiac maturation to CPCs, d22 CMs, and d60 CMs in both lines (Fig. 2e).

Since rescue experiments with pantethine and 4’-P-pantetheine in fibroblasts showed a stronger rescue effect on CoA level for pantethine, we treated iPSCs and iPSC-derived cardiac cells only with pantethine, which is generally available as a nutritional supplement. At the tested concentrations of 50, 150, and 500 µM, we recorded a substantial increase of CoA in both patients’ cells at all developmental stages (Fig. 2e). Western blotting did not reveal an increase in PPCS upon the treatment indicating that the observed increase in CoA is not due to stabilization of PPCS protein by pantethine (Fig. 2f).

### Pantethine mitigates PPCS DD phenotypes in patient-derived 2D and 3D cardiac models

Immunofluorescence analysis of the sarcomeric protein α-actinin revealed sarcomere disorganization in d60 CMs derived from both patient iPSC lines, suggesting a potential defect in contractile function, which was slightly rescued by treatment with 100 µM pantethine for 5 days (Fig. S7a, b). We then performed single-cell calcium (Ca^2+^) imaging using the fluorescent indicator Fluo-4 to visualize the intracellular Ca^2+^ dynamics underlying excitation-contraction coupling. Analysis of CMs under different electrical pacing conditions (0.4, 0.5, and 1 Hz) revealed significantly lower Ca^2+^ transient amplitudes in patient cells compared to controls, consistent with a cardiomyopathy phenotype, which was partially rescued by pantethine treatment (Fig. S7a, c, d). We also detected arrhythmic events in 42% of CMs from patient 95595 and 54% of CMs from patient 103596, which were reduced to 26% and 24%, respectively, by pantethine treatment (Fig. S7a, e).

**Fig. 3:**
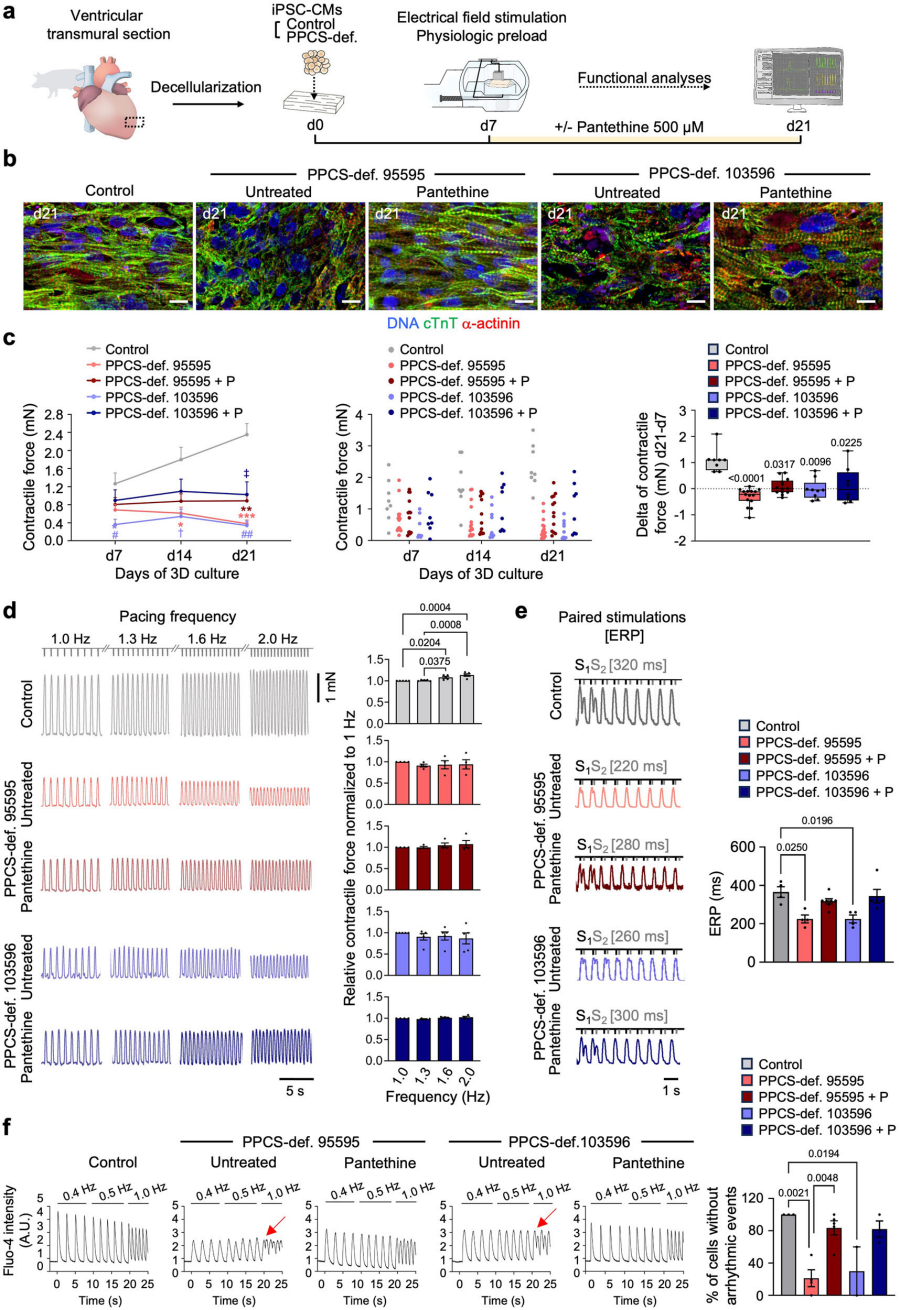
Pantethine ameliorates PPCS DD disease phenotypes in patient iPSC-derived 3D heart patches. **a** Heart patches were generated by seeding d15 CMs from control and PPCS deficient iPSCs (patients 95595 and 103596) onto decellularized extracellular matrix from porcine ventricular myocardium. After 7 days, recellularized patches were transferred to biomimetic chambers allowing continuous electromechanical stimulation; from this day on PPCS deficient patches were either untreated or treated with 500 µM pantethine until day 21 of 3D culture. **b** Immunofluorescence staining of cTnT and α-actinin at day 21 in control patches and PPCS deficient patches with or without pantethine. Scale bars = 10 µm. Images are representative of *n* = 3 patches per group. **c** (Left and middle) Contraction force of control patches and PPCS deficient patches with or without pantethine (P) on days 7, 14, and 21 of 3D culture. Mean ± SEM (left) and individual data points (middle), control (two lines): *n* = 8 patches; PPCS deficient 95595: untreated *n* = 15, + P *n* = 13 patches; PPCS deficient 103596: untreated *n* = 9, + P *n* = 8 patches. Two-way ANOVA with Tukey’s multiple comparisons test, d7: *p* = 0.0420 (#) PPCS deficient 103596 untreated vs control; d14: *p* = 0.0179 (*) PPCS deficient 95595 untreated vs control, *p* = 0.0138 (†) PPCS deficient 103596 untreated vs control; d21: *p* = 0.0003 (***) PPCS deficient 95595 untreated vs control, *p* = 0.0020 (**) PPCS deficient 95595 + P vs control; *p* = 0.0002 (##) PPCS deficient 103596 untreated vs control, *p* = 0.0230 (‡) PPCS deficient 103596 + P vs control. The source data are provided in the Supplementary Data 6. (Right) Corresponding delta of contractile force d21-d7. Box plots show all data points and indicate the median and 25th and 75th percentiles, with whiskers extending to the min and max values. Kruskal-Wallis test with Dunn’s multiple comparisons test, *p*-values vs control. **d** (Left) Representative traces of the contractile force of control patches and PPCS deficient patches with or without pantethine placed under increasing pacing frequencies (1.0, 1.3, 1.6, and 2.0 Hz) to assess their force-frequency relationship (FFR) on day 21 of 3D culture. (Right) Contractile force relative to 1 Hz, indicated as mean ± SEM. Control (two lines): *n* = 5 patches; PPCS deficient 95595: untreated *n* = 4, + P *n* = 4 patches; PPCS deficient 103596: untreated *n* = 5, + P *n* = 4 patches. Two-way ANOVA with Tukey’s multiple comparisons test. **e** (Left) Representative traces of the contractile force of control patches and PPCS deficient patches with or without pantethine submitted to paired stimulations at different intervals to determine their effective refractory period (ERP) on day 21 of 3D culture. (Right) ERP indicated as mean ± SEM. Control (two lines): *n* = 4 patches; PPCS deficient 95595: untreated *n* = 4, + P *n* = 7 patches; PPCS deficient 103596: untreated *n* = 4, + P *n* = 5 patches. Kruskal-Wallis test with Dunn’s multiple comparisons test. **f** (Left) Representative single-cell Ca^2+^ transients in control iPSC-CMs and PPCS deficient iPSC-CMs (patients 95595 and 103596) within patches with or without treatment with 500 µM pantethine, placed under 0.4, 0.5, and 1 Hz pacing frequencies. Red arrows indicate examples of arrhythmic events. (Right) Percentage of CMs without arrhythmic events, indicated as mean ± SEM; control (two lines): *n* = 3 independent differentiations; PPCS deficient 95595: untreated *n* = 4, + P *n* = 5 independent differentiations; PPCS deficient 103596: untreated *n* = 2, + P *n* = 3 independent differentiations. One-way ANOVA.

To further assess the functional features of PPCS deficiency, CMs in a 3D tissue-like context, d15 control and patient CMs were seeded onto decellularized extracellular matrix from porcine ventricular myocardium to generate 300 µm-thick heart patches, as previously described^24,26,34^ (Fig. 3a). To ensure consistent patch formation, we verified by flow cytometry that equivalent CM populations were obtained from control iPSCs and iPSCs from patients 95595 and 103596 (around 80% cTnT^+^ cells) (Fig. S8). A week after recellularization, the heart patches were placed in biomimetic chambers allowing continuous electromechanical stimulation and monitoring of contractile force^24,35^ (Fig. 3a).

While control CMs adopted an elongated shape aligned to the direction of contraction by day 21 of 3D culture, PPCS deficient CMs from both lines remained rather rounded with poor myofibril organization (Fig. 3b). In line with the 2D results, cell organization could be ameliorated by treatment with 500 µM pantethine from day 7 to 21 (Fig. 3b). Moreover, PPCS deficient patches from both lines showed a decrease of contractile force between days 7 and 21, contrasting with the significant increase of force indicating progressive tissue maturation in control patches (Fig. 3c). Pantethine treatment supported a stabilization of the contractile force, but no significant increase over time (Fig. 3c). Similarly, pantethine promoted an increase of the force-frequency relationship (FFR) of PPCS deficient patches at day 21, but it was not sufficient to achieve the positive FFR expected in healthy ventricular tissue^36^, which was observed in control patches (Fig. 3d). We then applied paired stimulations at decreasing time intervals to determine the effective refractory period (ERP) of patches, i.e., the interval during which no depolarization can be induced. PPCS deficient patches from both lines showed a significantly lower ERP at day 21 compared to control (Fig. 3e). Pantethine treatment markedly increased the ERP, albeit not to control levels (Fig. 3e). As a lower ERP can be indicative of increased arrhythmogenic potential, we additionally performed Ca^2+^ transient imaging in patches stimulated under different pacing conditions (0.4, 0.5, and 1 Hz). Indeed, consistent with our measurements in 2D, PPCS deficient patches from both lines showed a significantly higher occurrence of arrhythmic events than controls, which was markedly reduced by pantethine treatment (Fig. 3f).

## Discussion

The newly identified PPCS DD patients all present DCM as a constant finding, along with a spectrum of phenotypes including neuromuscular signs and neurologic deterioration. However their brain MRI were unremarkable, and no iron accumulation was observed, similar to previously reported cases^2,3^.

Despite the limited number of PPCS DD patients (*n* = 12), an exploratory genotype-phenotype correlation analysis suggests that the variants p.Ala20Gly, Tyr78His, Arg106Pro, Pro107_Ala111del, and Ala180Pro of the canonical PPCS correlate with DCM plus neuromuscular presentation, in contrast the p.Glu233Val variant leads to an isolated DCM presentation with variable expressivity, suggesting that different clinical presentations may be related to the position of the mutations within the gene. We hypothesize that the “DCM only” phenotype arises when variants involve exon 3, while “DCM plus” occurs when they involve exons 1 and 2 (Fig. 1b). Four alternative transcripts potentially encoding proteins are reported for PPCS (Fig. S9). The canonical form (ENSP00000361642) expresses all three exons, while the other forms are predicted to express a combination of exons 2 plus 3 (ENSP00000361643) or 1 plus 2 (ENSP00000361641), or have a completely different coding sequence (ENSP00000361637). We speculate that mutations affecting PPCS forms with different exon configurations, potentially expressed in a tissue- and time-specific manner, and localized to multiple cellular compartments, similar to the canonical protein, might contribute to the wide spectrum of clinical presentation of PPCS DD. For instance, fibroblasts, iPSCs and cardiac cells express the canonical PPCS form but not the ENSP00000361643, which shares the epitope of the antibody used in our Western blot analysis. This confirms that not all alternative isoforms are expressed ubiquitously. Clinical follow-up of Patient F3:III.3 who carries the variant p.Glu233Val, only expressed in the canonical form, and underwent heart transplant one year ago will help clarify the relevance of the canonical isoform for heart function. Although a long-term follow-up is needed, at one year post transplant, the patient has not experienced a recurrence of DCM, suggesting that the canonical isoform is primarily relevant for the heart’s metabolic and functional aspects.

Patients F1:II presented with neurological deterioration. Whether neurological involvement is part of the phenotypic presentation of PPCS DD or secondary to clinical interventions (i.e., ECMO) is difficult to ascertain, considering the very limited number of affected individuals reported so far. Only by investigating the natural history of the disease in a larger patient cohort and studying the consequence of PPCS deficiency specifically in neuronal cell models we will enable us to understand if and to what extent the brain is affected in this disorder.

Genetically, none of the new patients, similarly to previously reported cases, harbored biallelic loss of function variants, suggesting that this combination might be incompatible with life. Furthermore, all new variants and the reported Pro107_Ala111del variant^2^ hit exon 1, suggesting this genetic region as a mutational hotspot for PPCS.

All identified variants were predicted to be pathogenic. The Tyr78His variant changed an evolutionarily conserved amino acid relevant for the binding of PPCS substrate and the enzyme’s catalytic activity. In contrast, the variant Arg106Pro affects an amino acid relevant for the dimerization of PPCS, despite not being conserved.

Surprisingly, testing of Tyr78His and Arg106Pro variants in yeast, revealed no or moderate pathogenic effect, unlike the reported Pro107_Ala111del, Ala180Pro, and Glu233Val variants^2^. The absence of a phenotype in yeast could have different explanations: (i) Overexpressing human variants in yeast could lead to higher levels of PPCS transcript and protein (albeit with a variant) than those present in patient tissues, where variants destabilize RNA and reduce PPCS levels, as confirmed by the clinical transcriptome in patient F1:II. (ii) The stability of mutant PPCS forms in yeast might differ from that in mammalian cells. Although multiple proteolytic systems in yeast have human counterparts, the specific cleavage patterns may differ. (iii) The yeast complementation assay is qualitative, meaning that full complementation does not rule out mild enzymatic defects. For example, site-directed mutagenesis in the yeast pantothenate kinase (Cab1)^37^ has shown that a yeast enzyme with about 30-35% residual activity fully complements growth deficiency, while 25-30% residual activity results in some growth retardation, and activity below 20% causes growth deficiency.

To elucidate the effect of variants directly on patient samples, we performed WB experiments in fibroblasts. The analyses confirmed reduced PPCS level in all patient samples similar to reported cases^2,3^ and provided evidence that disease-relevant cell types (CPCs, CMs) and potentially all cell types (iPSCs) present with reduced protein.

Reduced protein levels were accompanied by reduced intracellular CoA, as previously reported^2,38^. Notably, undifferentiated iPSC cells showed a milder reduction of CoA compared to cells committed to the cardiac lineage, such as CPCs and CMs. This aligns with established evidence that iPSCs rely mainly on glycolysis for energy, while mature cardiac cells preferentially utilize fatty acids, requiring CoA for their activation and utilization^39^. Therefore, a shortage of CoA during cardiac cell maturation could lead to reduced energy supply for cardiac cells and cause cardiac dysfunction in PPCS DD patients.

Recent studies in fruit flies have highlighted that the gut microbiome can contribute to CoA homeostasis in the host by generating CoA from the precursor pantetheine (Fig. S1)^40^. Whether this cooperative mechanism exists in the mammalian gut and is not just peculiar to fruit flies, is unclear. If confirmed, part of the variable expressivity of PPCS DD among patients and siblings sharing the same pathogenic variants could be explained by differences in microbiome profile and dietary intake of CoA precursors in addition to the mutations variably affecting PPCS forms with different exon configurations. Notably, a few PPCS DD patients (F1:II, F2:II.1) developed DCM, or their condition worsened, after an infection requiring antibiotics, likely altering the gut microbiota. While the role of the microbiome in PPCS DD was not the focus of the current study, future investigations will explore the possibility that co-occurrence of pathogenic variants in PPCS and certain microbiome profiles predispose to variable expressivity of the disease. Therefore, sparing the microbiome from specific antibiotics or modulating the microbiome with diet or probiotics, might compensate for reduced de novo synthesis of CoA. Outcomes from these investigations might be relevant not only for PPCS DD, but also for PPCDC DD^4^ and for any other disease where a CoA deficit has been demonstrated.

Despite open questions about variable expressivity of PPCS DD, our results clarified important aspects of PPCS DD pathogenesis and treatment efficacy. We confirmed that reduced intracellular CoA is a reliable biomarker of PPCS DD, as not only fibroblasts^2,38^, but also disease-relevant cell types from patients such as CMs have reduced CoA.

Our study highlights the utility of patient-derived CMs and heart patches in elucidating the DCM phenotype associated with PPCS DD. Additionally, we showed that both pantethine and 4′-P-pantetheine can elevate intracellular CoA levels, with pantethine being slightly more effective. While 4′-P-pantetheine might be expected to be the preferred therapeutic candidate due to its greater stability in serum^19^, our results suggest that pantethine may be taken up more efficiently by the cells used in these experiments. The mechanism by which mammalian cells internalize molecules like pantethine and 4′-P-pantetheine remains unknown, making this an intriguing area for follow-up studies, particularly as it could inform the development of these molecules as therapeutic options.

Importantly, pantethine demonstrated the ability to improve or stabilize many aspects of the cardiomyopathy (i.e., sarcomere organization, Ca^2+^ amplitude, arrhythmia, FFR, ERP).

Therefore, even if full DCM reversal cannot be achieved by pantethine once cardiac damage has occurred, supplementation of pantethine throughout the maturation process from iPSCs to CMs and heart patches could eventually result in a complete rescue of the cardiomyopathy.

Overall, our data underline the importance of a timely diagnosis of PPCS DD and an early intervention with pantethine to prevent heart damage.

## Supplementary information

Supplementary Information

Supplementary Data Legends

Supplementary Data 1

Supplementary Data 2

Supplementary Data 3

Supplementary Data 4

Supplementary Data 5

Supplementary Data 6

Supplementary Data 7

Supplementary Data 8

Supplementary Data 9

Supplementary Data 10

Supplementary Data 11

Supplementary Data 12

Supplementary Data 13

Supplementary Data 14

Supplementary Data 15

Supplementary Data 16

Supplementary Data 17

Supplementary Data 18

Supplementary Data 19

Supplementary Data 20

Reporting Summary

## Data Availability

The numerical data plotted (source data) in the graphs in Fig. 2 are in Supplementary Data 1–5, in Fig. 3 are in Supplementary Data 6–10, in Fig. S2 are in Supplementary Data 11-12, in Fig. S5 are in Supplementary Data 13, in Fig. S6 are in Supplementary Data 14, in Fig. S7 are in Supplementary Data 15–19, and in Fig. S8 are in Supplementary Data 20.Other data used in this publication will be made available to qualified researchers who provide a valid research question within the scope of the studies, and may be subject to a data use agreement. Requests will be responded to within 30 days. Please direct inquiries to the corresponding authors (Arcangela Iuso, arcangela.iuso@helmholtz-munich.de and Alessandra Moretti amoretti@tum.de).
