# Supplementary material for: Pantethine ameliorates dilated cardiomyopathy features in PPCS deficiency disorder in patients and cell line models
Source: Commun Med (Lond). 2025 Jul 31;5:323. doi: 10.1038/s43856-025-01017-z (PMC12313872; doi:10.1038/s43856-025-01017-z)
Supplement: Supplementary file 1 — Supplementary Information [file 43856_2025_1017_MOESM1_ESM.pdf]

Supplementary Methods

Mutagenesis of *PPCS*

The *PPCS* variants were obtained by site-directed mutagenesis of the cloned *PPCS* ORF using the QuikChange II Site-Directed Mutagenesis kit (Stratagene) as reported in [1], using variant-specific primer pairs F:5′-TGGCTGTCCGCTCTGCCGCCTTCGGGCCCAGCCC-3′; R:5′-GGGCTGGGCCCGAAGGCGGCAGAGCGGACAGCCA-3′ to introduce the change 317G>C and F:5′-CTTCCTAGCCGCCGGCCACGGGGTCCTGTTCTTG-3′; R:5′-CAAGAACAGGACCCCGTGGCCGGCGGCTAGGAAG-3′ for the change 232 T>C. The following pLenti 6.3 plasmids were hence generated: PPCS_c.317 G>C and PPCS_c.232 T>C. PPCS_wild-type was generated previously [1].

Yeast complementation assay

Subcloning of *hPPCS* into the single-copy expression yeast plasmid p415-MET25 was performed as reported in [1], using the mammalian expression plasmids as templates.

Supplementary Results

Complementation analysis in yeast reveals minor pathogenicity of the newly identified variants

We performed functional complementation of the yeast *PPCS* (*yPPCS)* null background with the human wild-type *PPCS* and its putative pathogenic variants using the method of plasmid shuffling as reported in [1], considering that *yPPCS* (i.e., *CAB2*) is a gene essential for yeast viability [2]. To evaluate the functional relevance of identified *PPCS* variants, the genuine *yPPCS* gene was used as a positive control, while an empty vector was used as a negative control. **Figure S4** shows colony growth after 2 and 3 days, respectively. Complementation by variants *hPPCS* c.232 T>C (p.Tyr78His) is as effective as the *hPPCS* WT reference. In contrast, variant *hPPCS* c.317 G>C (p.Arg106Pro) clearly leads to reduced growth, nevertheless showing complementation substantially above the negative control. Variant *hPPCS* c.59 C>G (p.Ala20Gly) was not tested for complementation of the *yPPCS* null background.

Supplementary References

[1] Iuso A, Wiersma M, Schüller H-J, Pode-Shakked B, Marek-Yagel D, Grigat M, et al. Mutations in PPCS, Encoding Phosphopantothenoylcysteine Synthetase, Cause Autosomal-Recessive Dilated Cardiomyopathy. Am J Hum Genet 2018;102:1018–30. https://doi.org/10.1016/j.ajhg.2018.03.022.

[2] Olzhausen J, Schübbe S, Schüller H-J. Genetic analysis of coenzyme A biosynthesis in the yeast Saccharomyces cerevisiae: identification of a conditional mutation in the pantothenate kinase gene CAB1. Curr Genet 2009;55:163–73. https://doi.org/10.1007/s00294-009-0234-1.

Figure S1

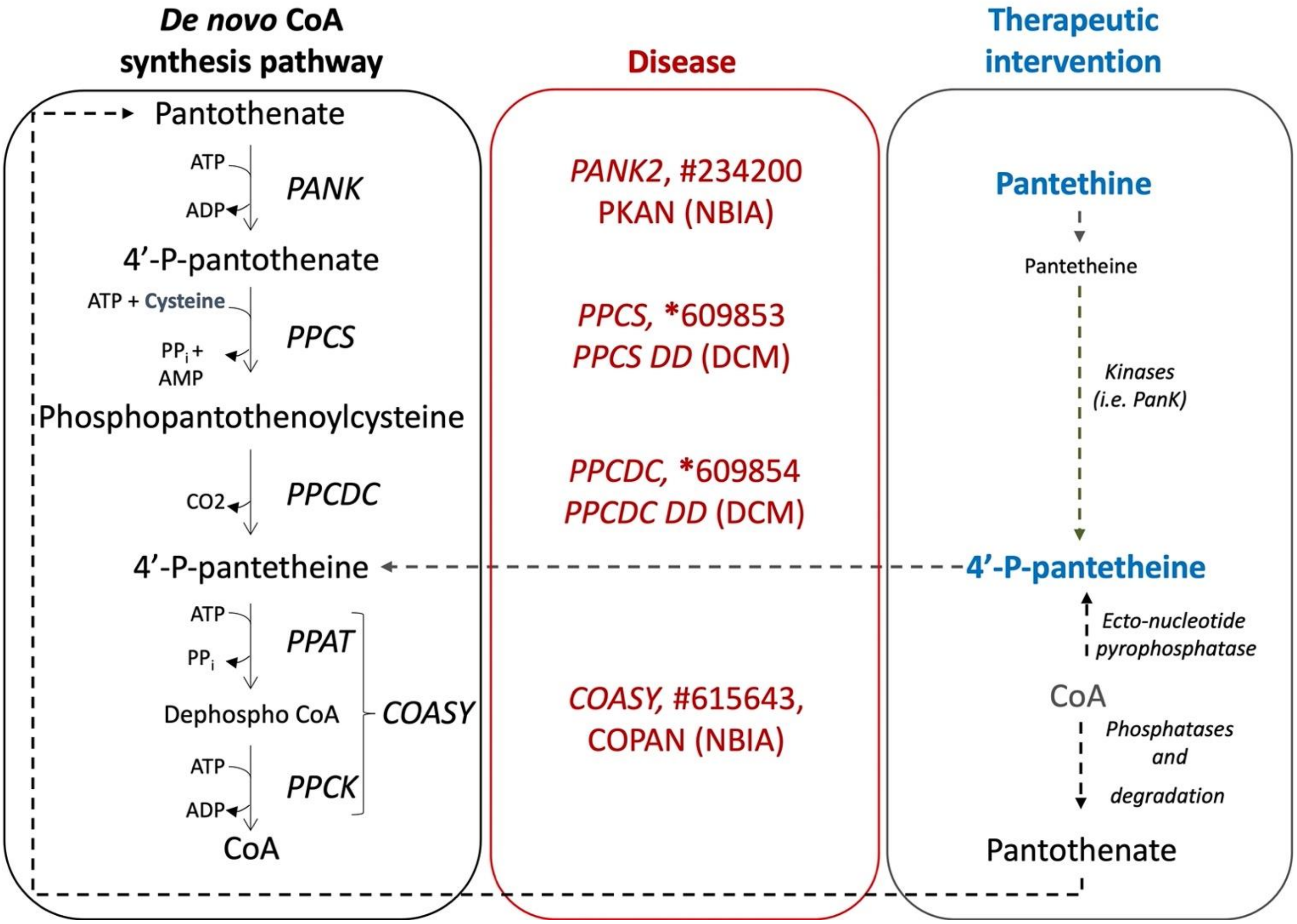

Figure S1 Universal pathway for the biosynthesis of CoA, associated diseases, and therapeutic interventions. Black dashed lines indicate the fate of CoA intermediates and their entry point in the pathway. NBIA: Neurodegeneration with Brain Iron Accumulation.

Figure S2

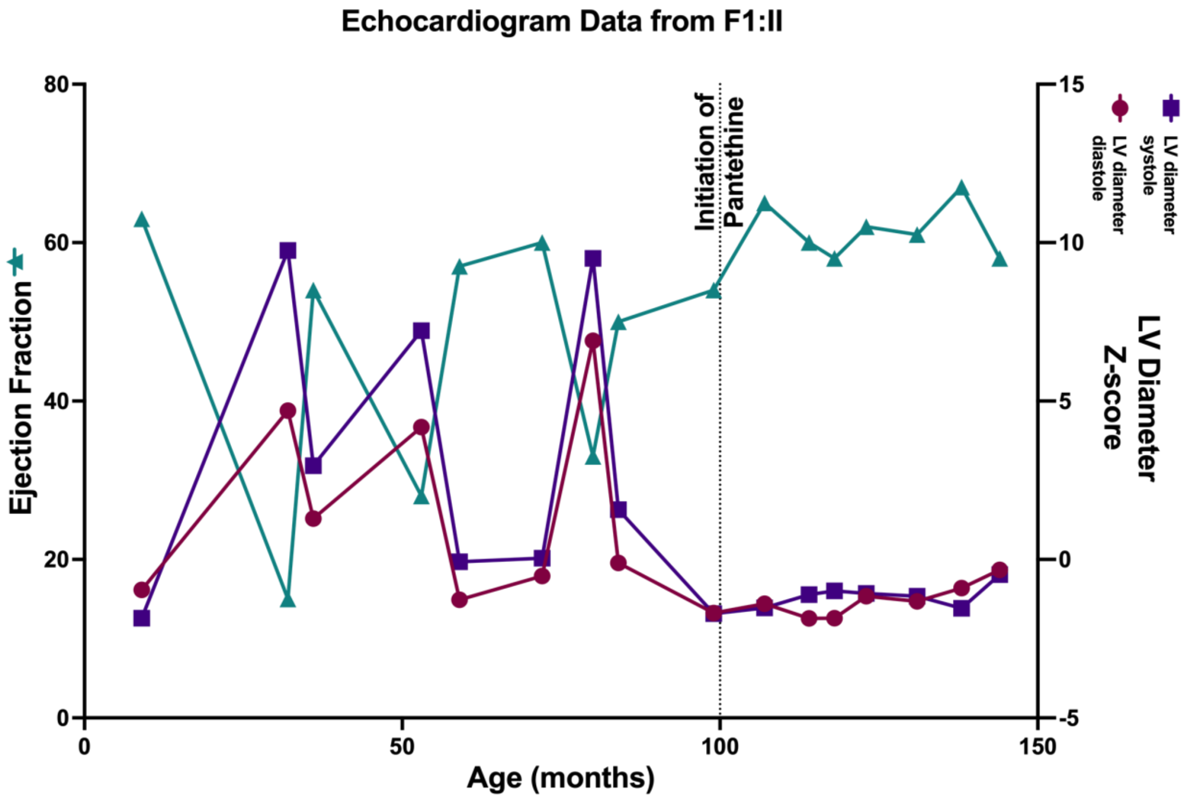
Echocardiogram Data from F1:II
Ejection Fraction
LV Diameter Z-score
LV diameter systole
LV diameter diastole
Initiation of Pantethine
Age (months)
80
60
40
20
0
15
10
5
0
-5
50
100
150

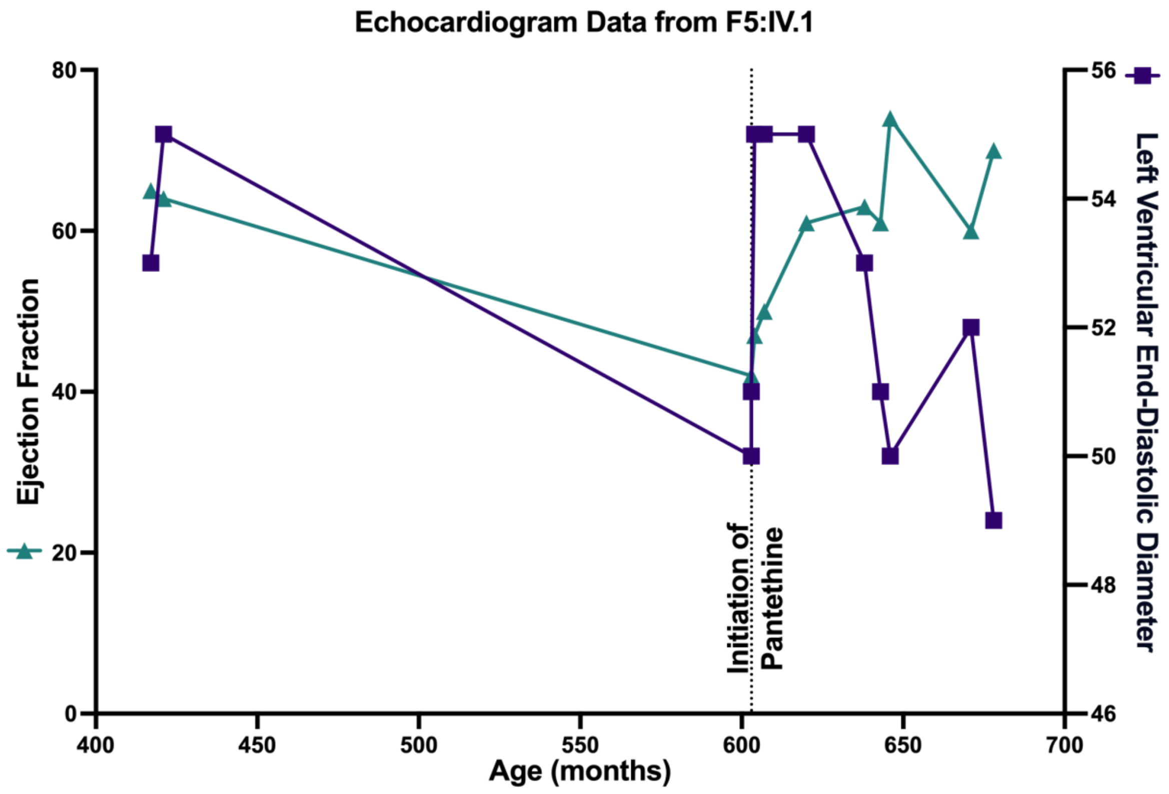
Echocardiogram Data from F5:IV.1
Ejection Fraction
Left Ventricular End-Diastolic Diameter
Initiation of Pantethine
Age (months)
80
60
40
20
0
56
54
52
50
48
46
400
450
500
550
600
650
700

Figure S2 Echocardiogram response to pantethine treatment in patients F1:II and F5:IV.1

The ejection fraction (left y-axis) and left ventricular (LV) diameter z-scores during diastole and systole (right y-axis) are shown for patient F1:II, with values recorded 100 months before the initiation of pantethine treatment and 50 months after treatment. The ejection fraction (left y-axis) and left ventricular end diastolic diameter (right y-axis) are shown for patient F5:IV.1, with values recorded 200 months before the initiation of pantethine treatment and 100 months after treatment.

Figure S3

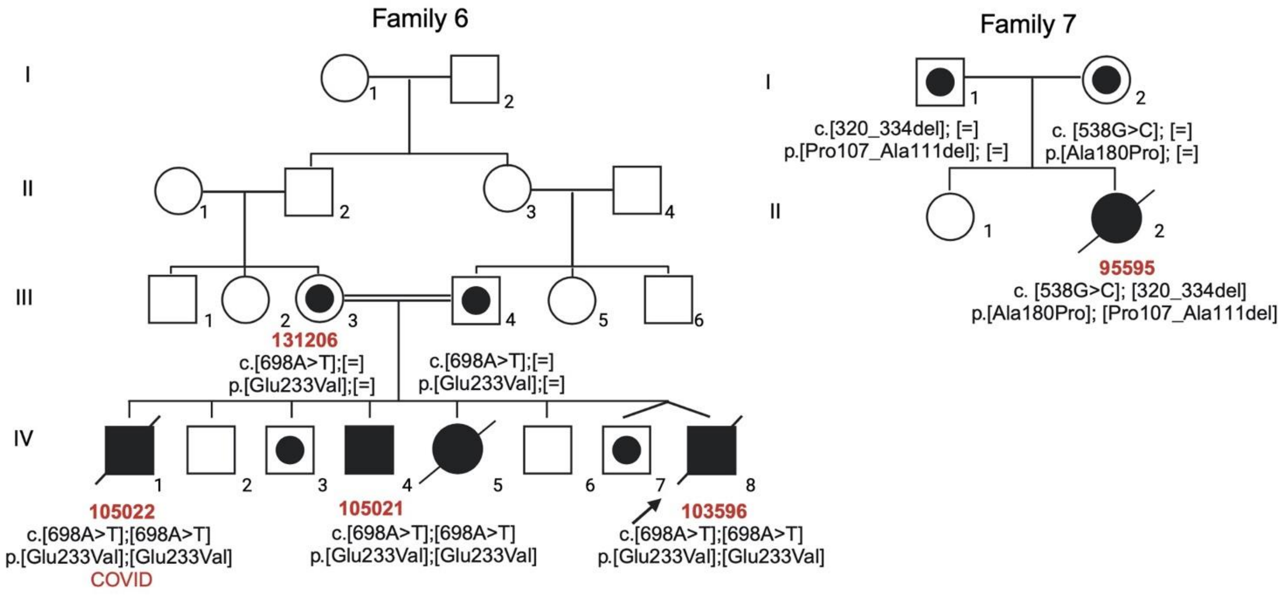

Figure S3 Pedigrees of two families with previously reported patients, whose fibroblasts were used to generate iPSCs, CPCs, and CMs. Affected individuals and healthy family members are indicated with closed and open symbols, respectively. Carriers are denoted by a dot in the center of the circle or square symbol. Fibroblast identification numbers are in red. Fibroblasts 103596 from F6:IV.8 and 95595 from F7:II.2 were used for follow up experiments in this study.

Figure S4

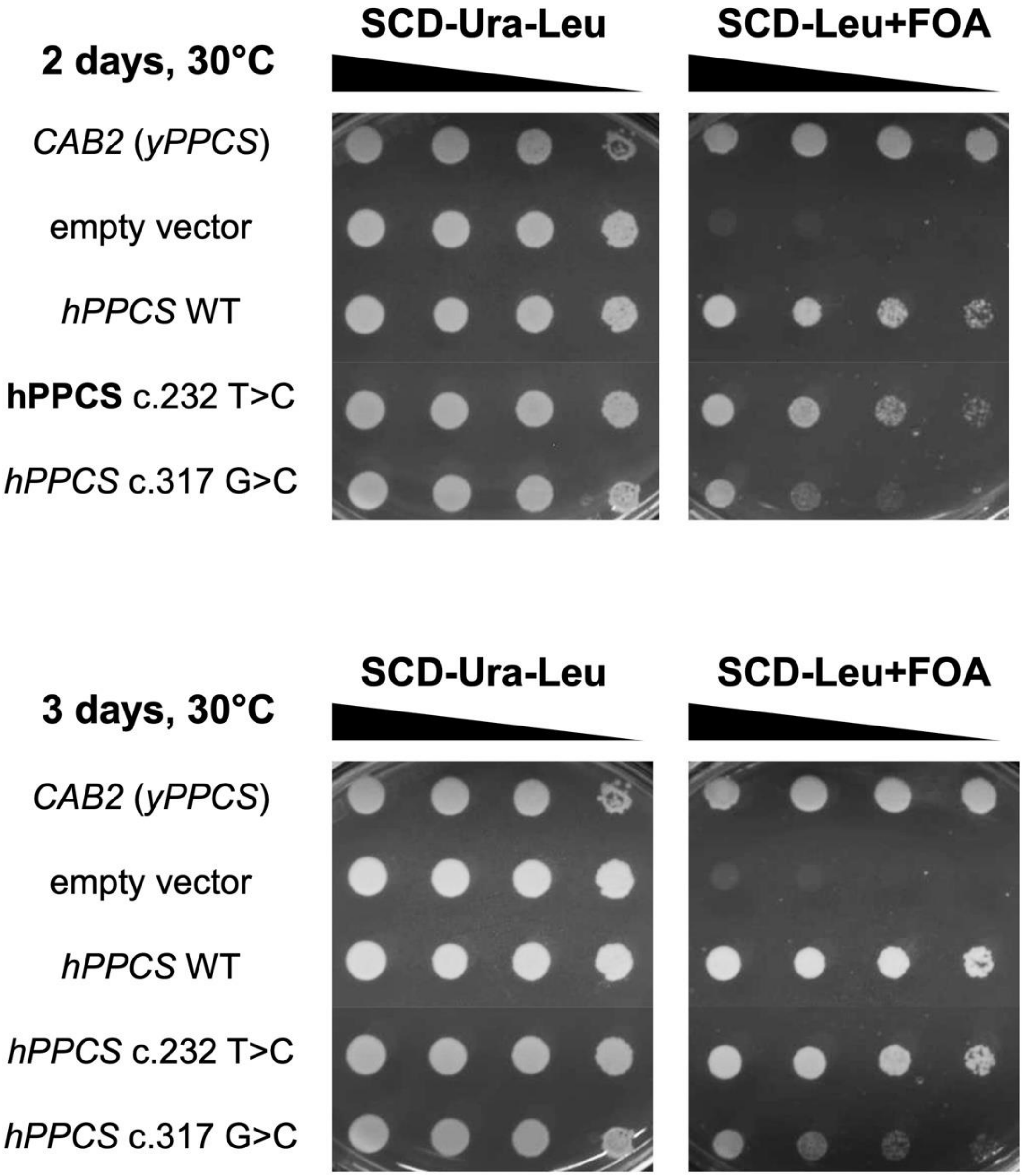

Figure S4 Functional complementation of a *S. cerevisiae cab2* deletion by plasmid shuffling experiments, using hPPCS variants. Strain MGY9 with a chromosomal deletion of the yeast PPCS gene (*CAB2*) contains single-copy rescue plasmid pGE11 (*URA3 CAB2*). Coding regions of hPPCS variants c.208 G>C, c.232 T>C and c.317 G>C were inserted into single-copy yeast expression plasmid p415-MET25, using the *MET25* promoter for heterologous gene expression. The resulting plasmids were transformed into MGY9 (selecting on SCD-Ura-Leu, left) and subsequently transferred on medium supplemented with 5-FOA for two or three days (SCD-Leu+FOA, right). Since 5-FOA counter-selects rescue plasmid pGE11, viability of the strain is dependent on the function of hPPCS variants.

Figure S5

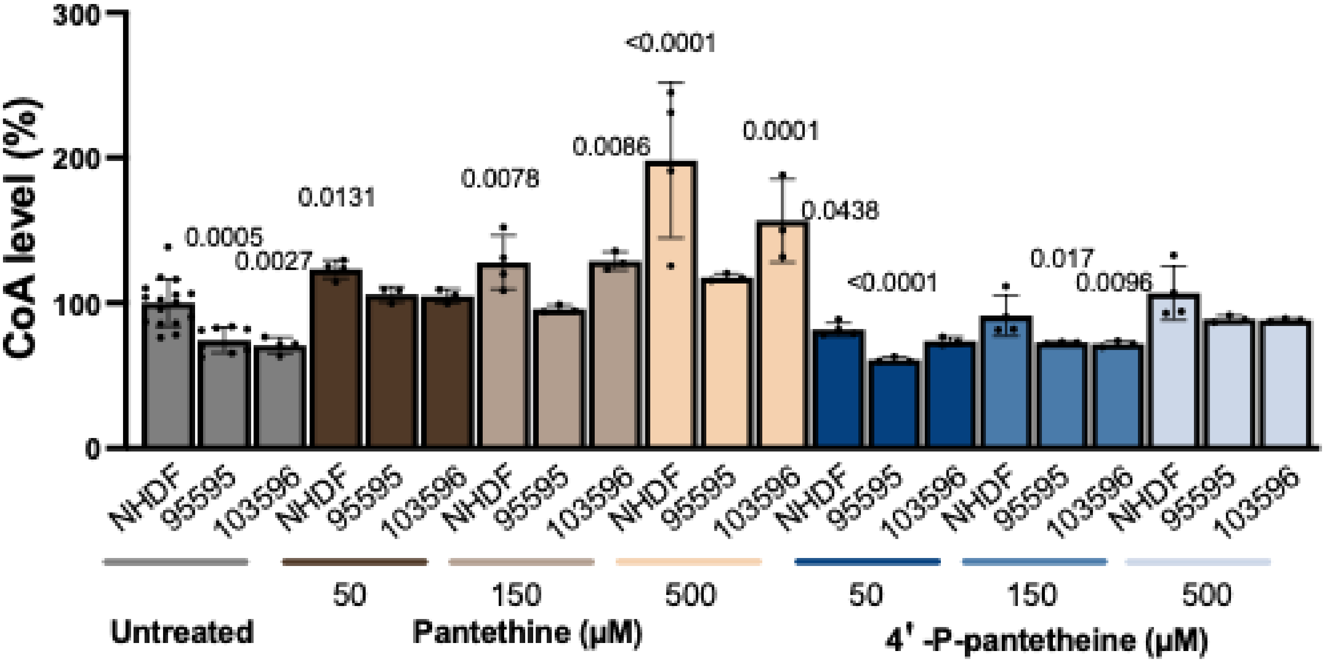

Figure S5 Pantethine and 4'-P-pantetheine restore CoA levels in fibroblasts from individuals with PPCS DD. Intracellular levels of CoA in fibroblasts from a healthy control subject (NHDF) and PPCS DD patients (95595,103596). Measurements were performed in standard growth conditions (untreated) and in presence of 50, 150 and 500 µM pantethine and 4'-P-pantetheine. The levels of CoA in the untreated healthy control were set to 100 and levels in the samples express as percent (%) of the untreated control value. Data are mean ± SD of n = 3 independent experiments. Statistical significance relative to the untreated control was determined using an independent samples t-test, with exact p-values reported.

Figure S6

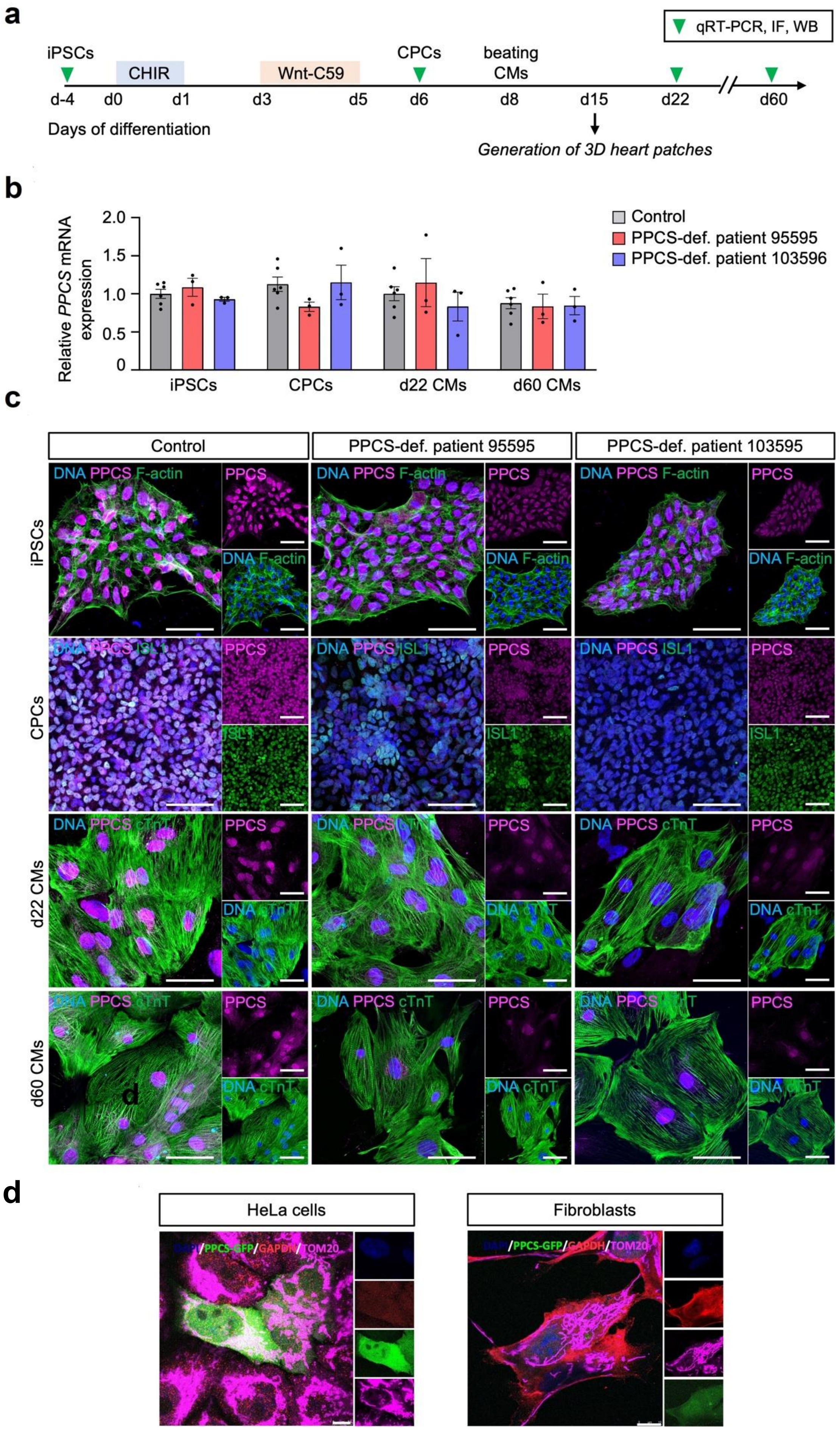
a
qRT-PCR, IF, WB
iPSCs
CHIR
Wnt-C59
CPCs
beating CMs
d-4
d0
d1
d3
d5
d6
d8
d15
d22
d60
Days of differentiation
Generation of 3D heart patches
b
Relative PPCS mRNA expression
2.0
1.5
1.0
0.5
0
iPSCs
CPCs
d22 CMs
d60 CMs
Control
PPCS-def. patient 95595
PPCS-def. patient 103596
c
Control
PPCS-def. patient 95595
PPCS-def. patient 103595
iPSCs
CPCs
d22 CMs
d60 CMs
DNA PPCS F-actin
PPCS
DNA F-actin
DNA PPCS ISL1
ISL1
DNA PPCS cTnT
DNA cTnT
d
HeLa cells
Fibroblasts
DAPI/PPCS-GFP/GAPDH/TOM20

Figure S6 Expression of *PPCS* in cardiac cells derived from PPCS patient iPSCs and cellular localization of PPCS. (a) WNT modulation protocol used for the differentiation of iPSCs into cardiac progenitor cells (CPCs) and cardiomyocytes (CMs). Green arrowheads indicate the developmental stages at which cells were collected for quantitative real-time PCR (qRT-PCR), immunofluorescence (IF), and western blot (WB) analyses. CMs were collected on d15 for the generation of 3D heart patches. CHIR: CHIR99021; d: day. **(b)** qRT-PCR analysis of *PPCS* mRNA expression in control and PPCS deficient iPSCs (patients 95595 and 103596) and derived CPCs, d22 CMs, and d60 CMs. Expression levels normalized to *GAPDH*, indicated as mean ± SEM, control (two lines): n = 6, PPCS deficient 95595 and 103596: n = 3 independent differentiations per line. **(c)** IF staining of PPCS together with F-actin, ISL1, or cTnT in control and PPCS deficient iPSCs (patients 95595 and 103596) and derived CPCs, d22 CMs, and d60 CMs. Scale bars = 50 µm. Images are representative of n = 2 independent differentiations. **(d)** PPCS (GFP, green) and nucleus (DAPI, blue) fluorescent signals together with ICC staining of Tom20 and GAPDH. Scale bar = 75 µm for HeLa cells and 10 µm for fibroblasts. Images are representative of n = 2 independent transfection experiments for cell type.

Figure S7

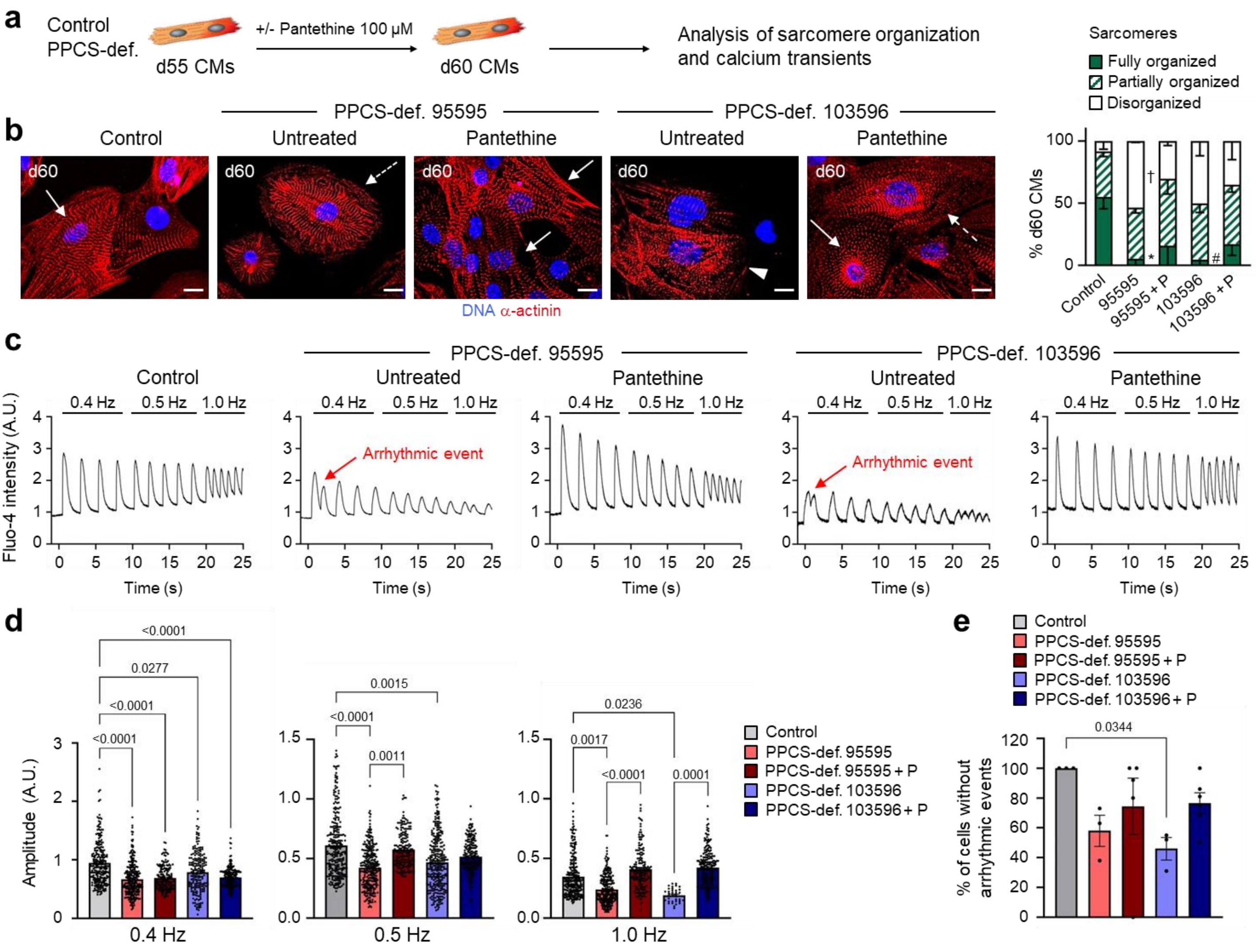

Figure S7 Pantethine ameliorates PPCS DD disease phenotypes in patient iPSC-derived cardiomyocytes. (a) d60 Control and PPCS deficient iPSC-CMs (with and without treatment with 100 μM pantethine for 5 days) were used for the analysis of sarcomere organization and calcium measurements. **(b)** (Left) Representative immunofluorescence staining of cTnT (green) and α-actinin (red) labeling sarcomere Z-discs in d60 control iPSC-CMs and PPCS deficient iPSC-CMs (patients 95595 and 103596) with or without treatment with 100 μM pantethine from days 55 to 60. Scale bars = 10 μm. Solid and dashed arrows indicate cells with fully (more than 90% of myofibrils are cross-striated) and partially (ca. 50-90% of myofibrils are cross-striated) organized sarcomeres, respectively. Arrowhead marks a cell with disorganized (less than 50% of myofibrils are cross-striated) sarcomeres. (Right) Corresponding percentage of CMs with fully and partially organized as well as disorganized sarcomeres, indicated as mean ± SEM; n = 3 (control, two lines, 102 cells) and n = 2 (PPCS deficient 95595 untreated: 489 cells, treated: with pantethine (+P) 257 cells; PPCS deficient 103596 untreated: 314 cells, treated with pantethine: (+P) 267 cells) independent differentiations.

Two-way ANOVA with Sidak's multiple comparisons test, fully organized sarcomeres: $p = 0.0141$ (*) PPCS deficient 95595 vs control, $p = 0.0124$ (#) PPCS deficient 103596 vs. control; disorganized sarcomeres: $p = 0.0332$ (†) PPCS deficient 95595 vs control. **(c-e)** (**c**) Representative single-cell Ca^{2+} transients in d60 control iPSC-CMs and PPCS deficient iPSC-CMs (patients 95595 and 103596) with or without treatment with 100 µM pantethine (P) from days 55 to 60, placed under 0.4, 0.5, and 1 Hz pacing frequencies. Arrows indicate examples of arrhythmic events. (**d**) Amplitude of Ca^{2+} transients at 0.4, 0.5, and 1 Hz pacing frequencies, indicated as mean ± SEM. For control (two lines) 0.4 Hz: n = 239 transients in 55 cells, 0.5 Hz: n = 268 transients in 54 cells, 1.0 Hz: n = 268 transients in 54 cells; for PPCS deficient 95595 untreated 0.4 Hz: n = 260 transients in 56 cells, 0.5 Hz: n = 272 transients in 55 cells, 1.0 Hz: 265 transients in 44 cells, treated with pantethine (+ P) 0.4 Hz: n = 173 transients in 43 cells, 0.5 Hz: 176 transients in 43 cells, 1.0 Hz: 167 transients in 39 cells; for PPCS deficient 103596 untreated 0.4 Hz: n = 192 transients in 45 cells, 0.5 Hz: n = 287 transients in 61 cells, 1.0 Hz: 36 transients in 9 cells, treated with pantethine (+ P) 0.4 Hz: n = 208 transients in 50 cells, 0.5 Hz: 213 transients in 50 cells, 1.0 Hz: 206 transients in 51 cells. Nested one-way ANOVA with Tukey's multiple comparisons test. (**e**) Percentage of CMs without arrhythmic events, indicated as mean ± SEM; control (two lines): n = 3 independent differentiations; PPCS deficient 95595: untreated n = 3, +P n = 5 independent differentiations; PPCS deficient 103596: untreated n = 3, +P n = 5 independent differentiations. Kruskal-Wallis test.

Figure S8

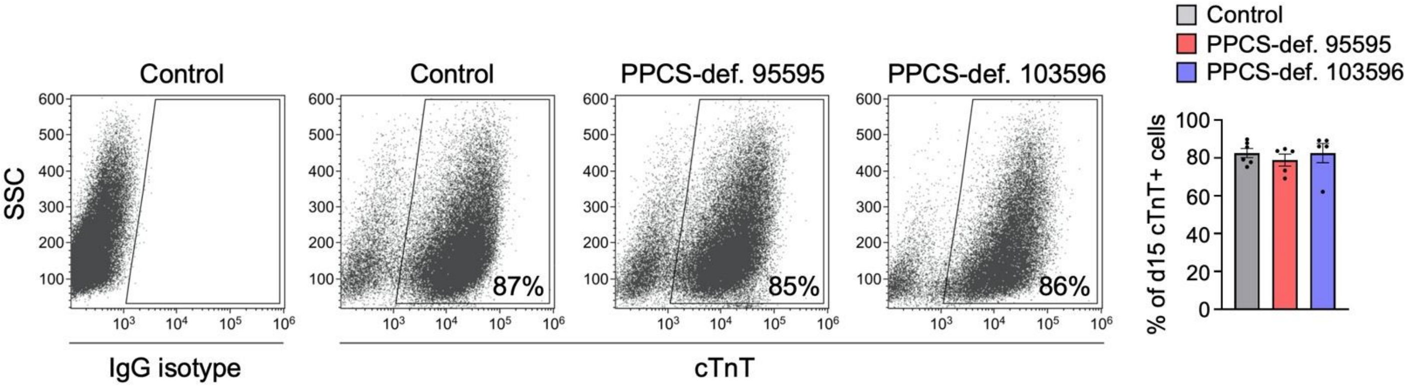

Figure S8 Validation of CM differentiation efficiency for the generation of heart patches. (Left) Representative plots of flow cytometry analysis of the CM marker cTnT and an IgG isotype control on d15 of cardiac differentiation of control and PPCS deficient iPSCs (patients 95595 and 103596). Side scatter (SSC) was used to measure cell granularity and internal complexity. The percentage of $cTnT^{+}$ cells is indicated. (Right) Percentage of $cTnT^{+}$ cells obtained from each line, indicated as mean ± SEM. Control (two lines): n = 6 independent differentiations, 95595: n = 5 independent differentiations; 103596: n = 5 independent differentiations.

Figure S9

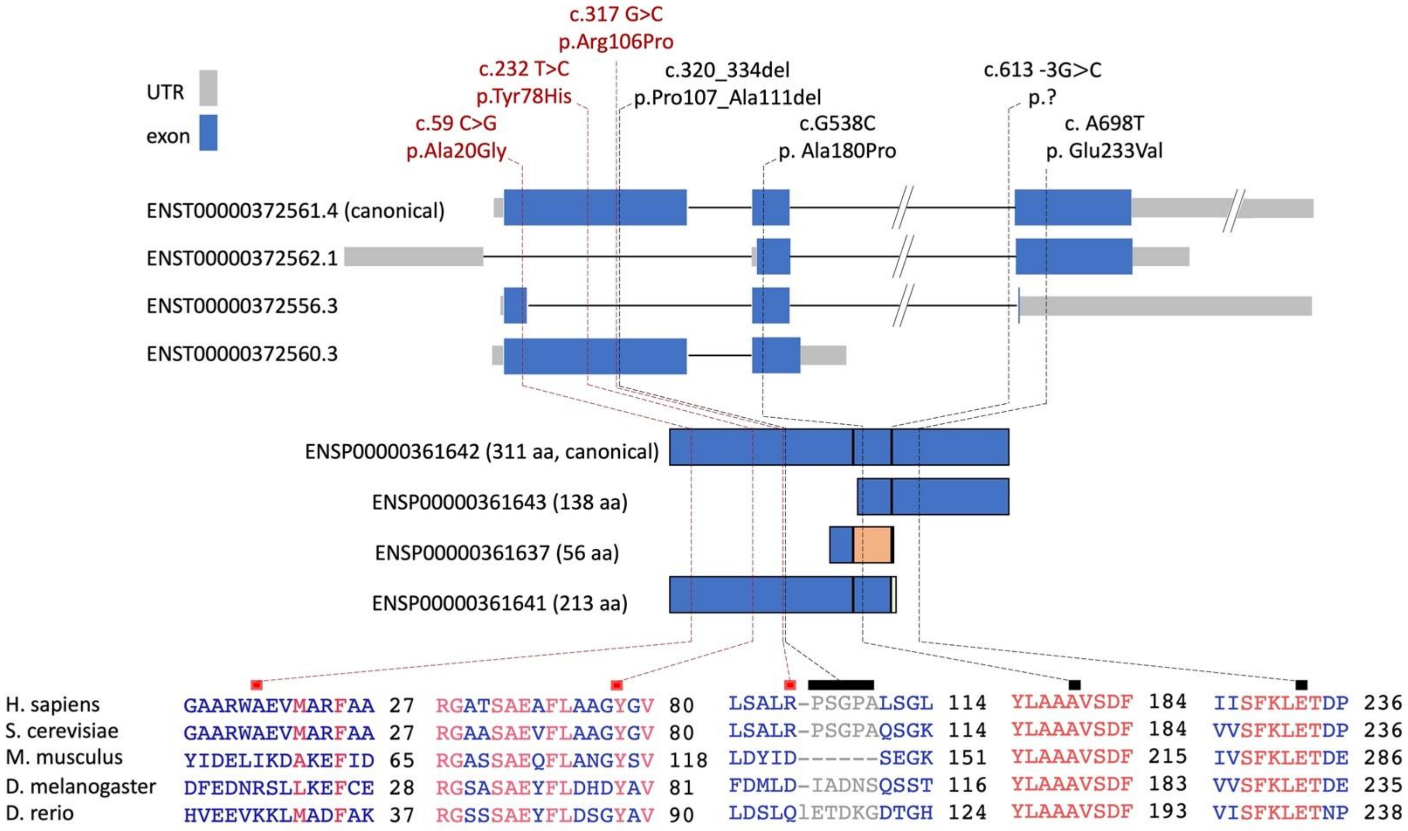

Figure S9 Alternative *PPCS* transcripts potentially encoding proteins. Localization of the newly identified (red) and already reported (black) pathogenic variants in PPCS at the gene, canonical and alternative transcript and protein levels, with a zoom in the conservation of amino acid residues affected by mutations. Coloring in the sequence alignment represents the identity of amino acid residues (COBALT alignment tool). Scale gene: 100 bp = 1 cm; scale proteins: 1 cm = 33 amino acids.

Figure S10

PPCS, tubulin, and ladder overlay

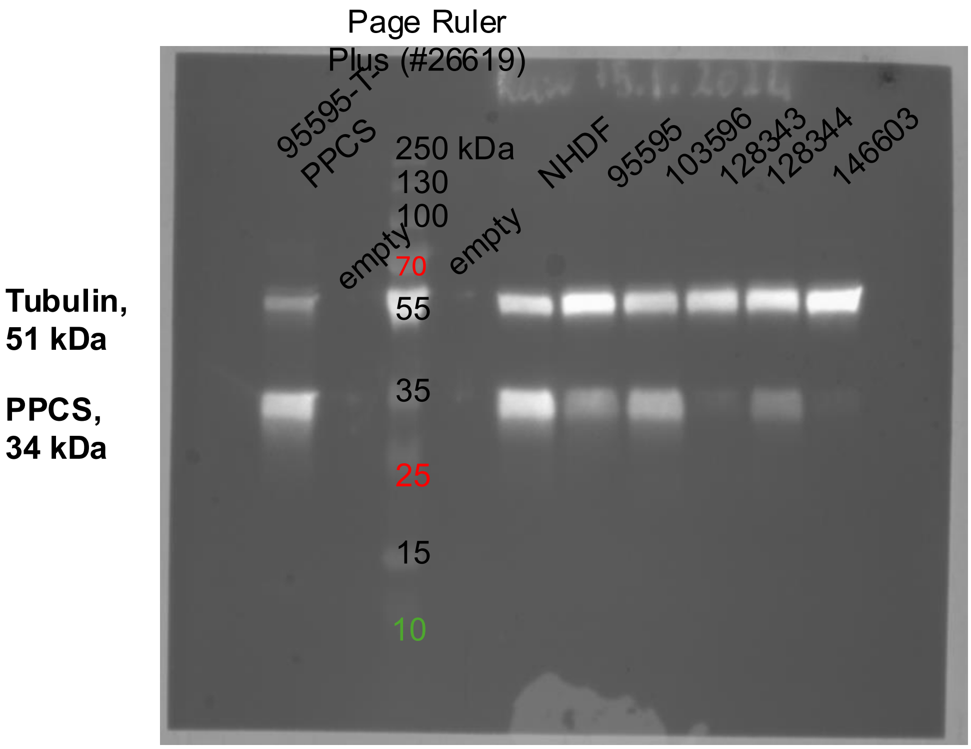

Invert, no ladder overlay, crop

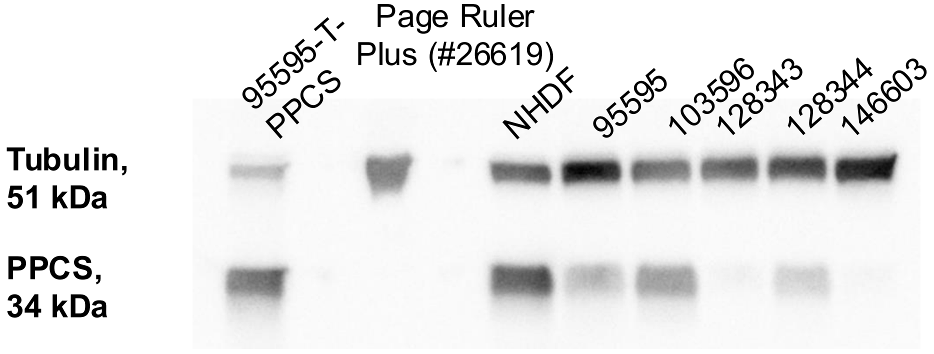

Figure S10 Uncrop Figure 2a

Figure S11

Ponceau staining

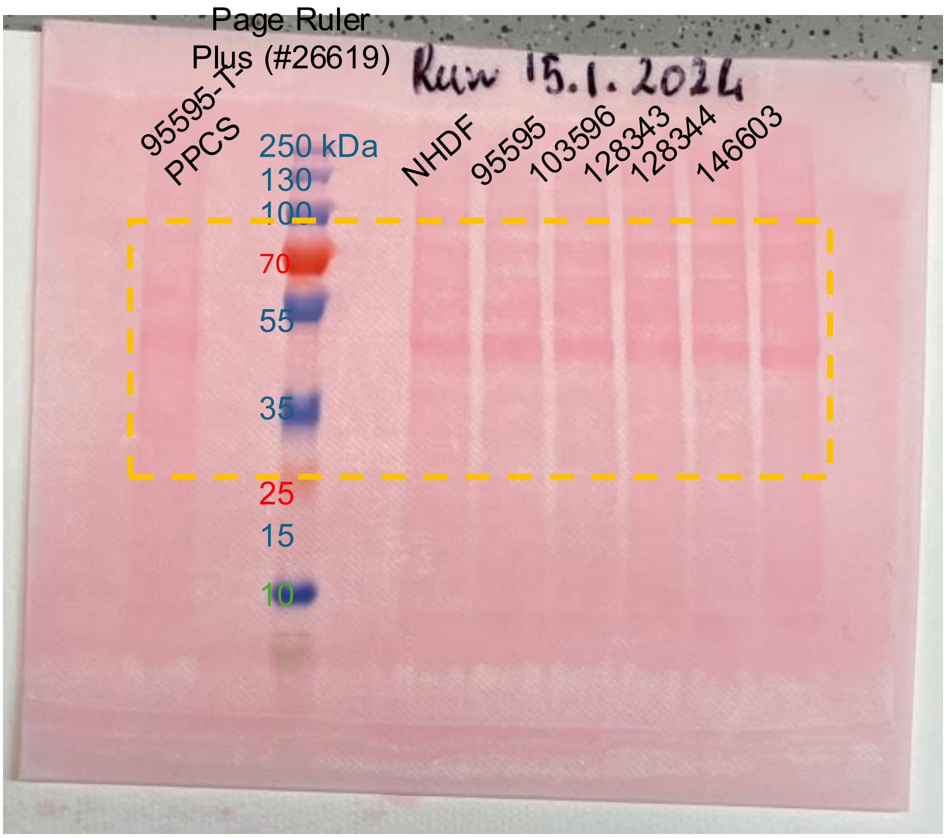

Crop

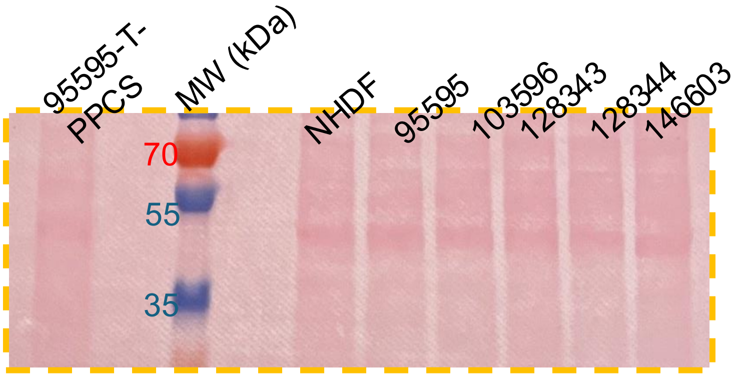

Figure S11 Uncrop Figure 2b

Figure S12

PPCS and ladder overlay

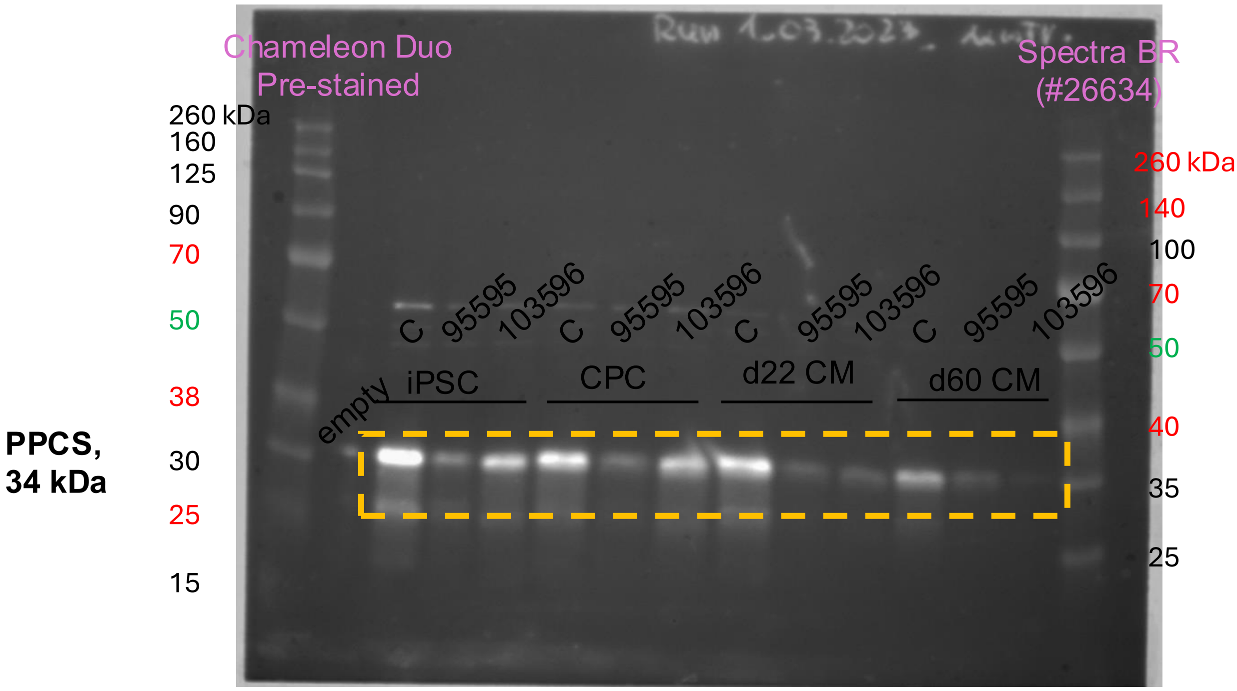

Actin and ladder overlay

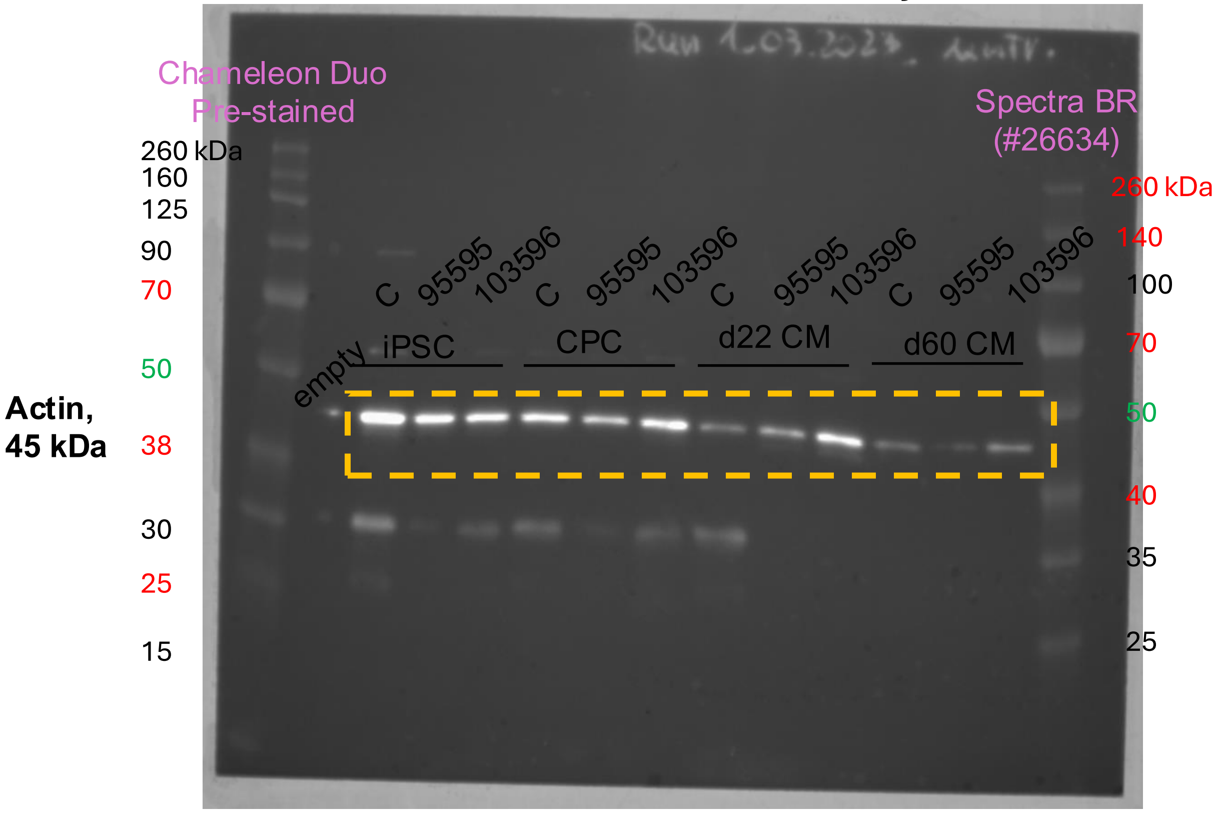

Invert, no ladder overlay, crop

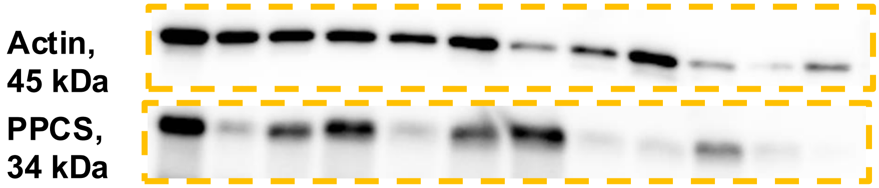

Figure S12 Uncrop Figure 2d

Figure S13

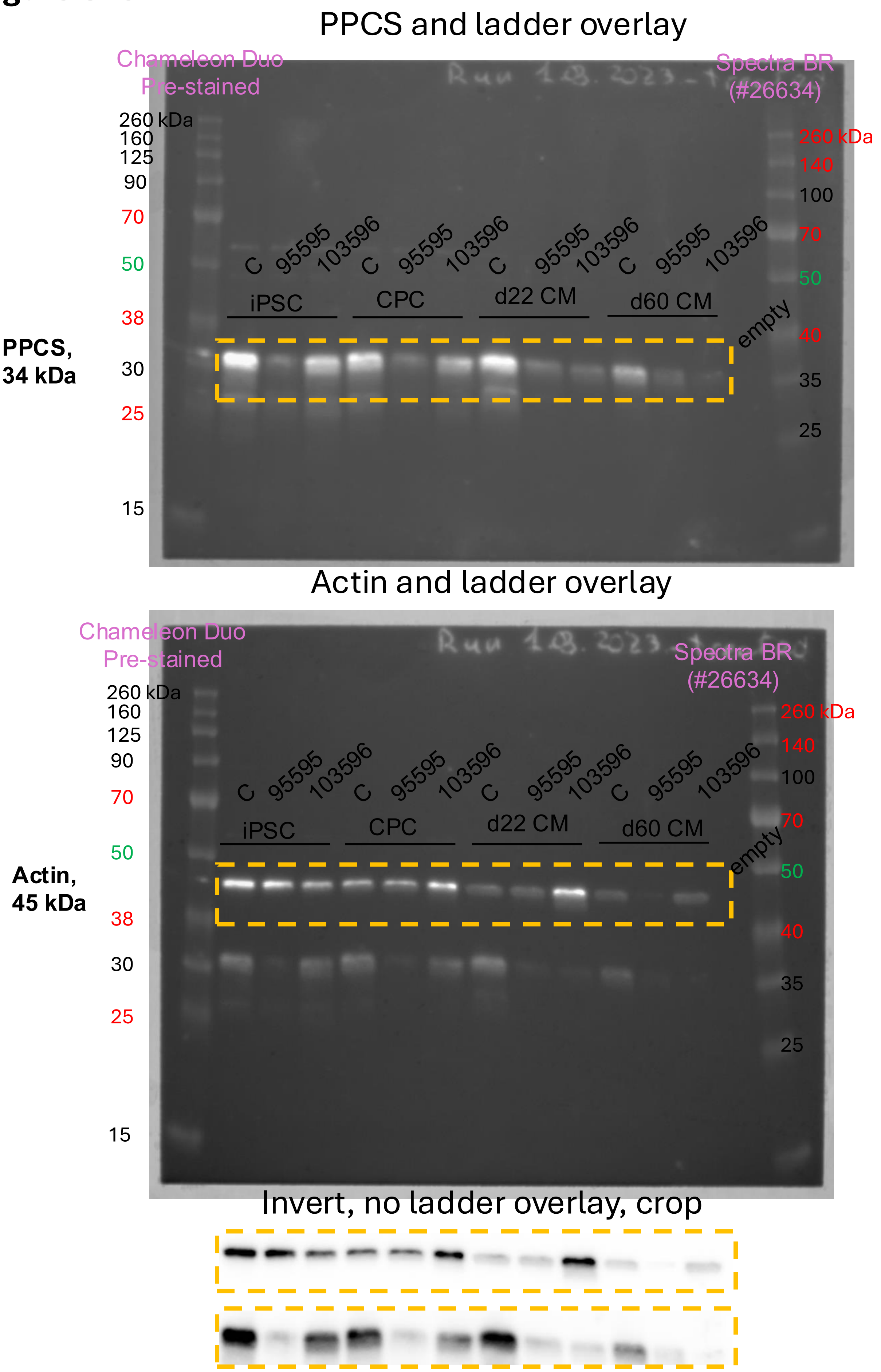

Figure S13 Uncrop Figure 2f

Table S1. List of all patients described in this study, including their baseline characteristics before pantethine intervention and outlining the pantethine treatment they received (dose, duration, tolerability.). LV: left ventricle, EJ: ejection fraction, CK: creatine kinase

Individuals described in this study	Patient ID	Mutation in PPCS	Baseline characteristics	Pantethine treatment	Treatment outcome
Case 1	F1:II	c.[232T>C]; [232 T>C] p.[Tyr78His];[Tyr78 His]	Cardiac symptoms: Severe DCM and cardiomegaly. Variable episodes of declining EF and prolonged QT; Extra cardiac symtpoms: skeletal muscle pain, weakness rhabdomyolysis; transient elevations of multiple long-chain fatty acyl-CoA species, lactic aciduria, ketonuria; upper respiratory symptoms; poor feeding; global loss of parenchymal brain volume with prominent sulci and ventricles, scattered nonspecific foci of T2 prolongation in the subcortical and deep white matter	Treatment dose: 450 mg per day (17 mg per kg per day) for 1 year, then increased to 900 mg per day starting in the second year. Total treatment duration: 3 years. Well tolerated. Excretion of 3-methylcrotonylglycine in the urine following pantethine treatment	Resolution of rhabdomyolysis along with improved appetite and energy levels. Normalization of the LV size, with function at the lower end of the normal range
Case 2	F2:II.1	c.[317G>C]; [317G>C] p.[Arg106Pro];[Arg106Pro]	Cardiac symptoms: Severe DCM with LV dilation, and declining EF. Extra cardiac symptoms: upper respiratory congestion and shortness of breath; metabolic acidosis; elevated CK levels	-	-
Case 3	F3:III.2	c.[698A>T]; [698A>T] p.[Glu233Val];[Glu233Val]	LV non-compaction cardio myopathy, with sinus tachycardia, low voltage QRS, and T-wave inversion in inferolateral leads, mildly diminished LV systolic function and increased LV trabeculations	-	-
Case 4	F3:III.3	c.[698A>T]; [698A>T] p.[Glu233Val];[Glu233Val]		-	-
Case 5	F4:II	c.[59C>G];[59C>G] p.[Ala20Gly];[Ala20 Gly]	Cardiac symptoms: Severe DCM, multiple episodes of near syncope, recurrent ventricular arrhythmias, mild dilation of the LV and moderate to severely depressed LV systolic function	-	-
Case 6	F5:IV.1	c.[698A>T]; [698A>T] p.[Glu233Val];[Glu233Val]	Cardiac symptoms: Transitory prolonged QT interval, septal hypertrophy, dilated LV with severe biventricular dysfunction with LV ejection fraction. Extra cardiac symptoms: rhabdomyolysis, liver dysfunction; mild hypokalemia and hypomagnesemia	Treatment dose: 600 mg/day (10 mg/kg/day). Total treatment duration: 1 year. Well tolerated	Resolution of the arrhythmia; full recovery of the LV function
Case 7	F6:IV.1 (FB:IV.1 in PMID 29754768)	c.[698A>T]; [698A>T] p.[Glu233Val];[Glu233Val]	Cardiac symptoms: DCM with mildly dilated LV with moderate to severe decrease in function of both ventricles; EF of 36% and exertional dyspnea	Treatment dose: 15 mg/kg/day. Total treatment duration: 4 years. Well tolerated	Stabilization of the clinical condition. EF improved and levelled off to 48%
Case 8	F6:IV.4 (FB:IV.4 in PMID 29754768)	c.[698A>T]; [698A>T] p.[Glu233Val];[Glu233Val]	Cardiac symptoms: DCM with an EF of 37%. Extra cardiac symptoms: increased long chain acylcarnitines, abnormal urine organic acid profile; easy fatigability, limited exercise tolerance	Treatment dose: 15 mg/kg/day Total treatment duration: 6 years. Well tolerated	Stabilization of the clinical condition. EF improved and levelled off to 45%

Table S2. List of the cell lines generated from patients and used in this study.

Patient ID	Mutation in PPCS	Fibroblast ID	Stably transduced fibroblasts	iPSCs ID	NPC, CMs, 3D heart patches
F1:II	c.[232 T>C]; [232 T>C] p.[Tyr78His];[Tyr78His]	128343	-	-	-
F2:II.1	c.[317G>C]; [317G>C] p.[Arg106Pro];[Arg106 Pro]	128344	-	-	-
F4:II	c.[59C>G];[59C>G] p.[Ala20Gly];[Ala20Gl y]	146603	-	-	-
F6:IV.8 (FB:IV.8 in PMID 29754768)	c.[698A>T]; [698A>T] p.[Glu233Val];[Glu233 Val]	103596	-	103596 (MRIi028-A)	103596
F7:II.2 (FA:II.2 in PMID 29754768)	c.[538G>C]; [320_334del] p.[Ala180Pro]; [Pro107_Ala111del]	95595	95595-T-PPCS	95595 (HMGUi003-A)	95595

Table S3. List of all patients previously identified with mutations in PPCS, including references (PMID) for where their clinical presentations were reported, outlining the pantethine treatment they received and outcomes that were already reported. EF: Ejection fraction.

Individuals reported in the literature	Mutation in PPCS	PMID (Patient ID)	Pantethine treatment	Treatment outcome
Case 1	c.[698A>T]; [698A>T] p.[Glu233Val];[Glu233Val]	29754768 (FB:IV.1)	Treatment dose: starting at 6 mg/kg/day gradually increased to 24 mg/kg/day over 8 weeks. Treatment duration: 12 months at the time of publication.	Mild improvement in exertional dyspnea. The EF (Simpson mode) increased from 36% at baseline to 48%.
Case 2	c.[698A>T]; [698A>T] p.[Glu233Val];[Glu233Val]	29754768 (FB:IV.4)	Treatment dose: starting at 6 mg/kg/day gradually increased to 24 mg/kg/day over 8 weeks. Treatment duration: 12 months at the time of publication.	Stabilization of the clinical condition with symptoms of heart failure only during exertion. The EF (Simpson mode) increased from 37% at baseline to 45%.
Case 3	c.[698A>T]; [698A>T] p.[Glu233Val];[Glu233Val]	29754768 (FB:IV.5)	-	-
Case 4	c.[698A>T]; [698A>T] p.[Glu233Val];[Glu233Val]	29754768 (FB:IV.8)	-	-
Case 5	c.[538G>C]; [320_334del] p.[Ala180Pro]; [Pro107_Ala111del]	29754768 (FA:II.2)	-	-
Case 6	c.[613-3C>G]; [320_334del] p.[?]; [Pro107_Ala111del]	35616428	-	-
