# Supplementary material for: Pantethine ameliorates dilated cardiomyopathy features in PPCS deficiency disorder in patients and cell line models
Source: Commun Med (Lond). 2025 Jul 31;5:323. doi: 10.1038/s43856-025-01017-z (PMC12313872; doi:10.1038/s43856-025-01017-z)
Supplement: Supplementary file 2 — Supplementary Data Legends [file 43856_2025_1017_MOESM2_ESM.pdf]

Supplementary Data Legends

Supplementary Data 1. Figure 2c data
Supplementary Data 2. Figure 2e_CPC data
Supplementary Data 3. Figure 2e_d22 CM data
Supplementary Data 4. Figure 2e_d60 CM data
Supplementary Data 5. Figure 2e_iPSC data
Supplementary Data 6. Figure Figure 3c_contraction_forcedata
Supplementary Data 7. Figure 3c_delta_contraction_forcedata
Supplementary Data 8. Figure 3d data
Supplementary Data 9. Figure 3e data
Supplementary Data 10. Figure 3f data
Supplementary Data 11. Figure S2_ECHO Data-F1 II data
Supplementary Data 12. Figure S2_ECHO Data-F5_IV data
Supplementary Data 13. Figure S5 data
Supplementary Data 14. Figure S6b
Supplementary Data 15. Figure S7b
Supplementary Data 16. Figure S7d_0.4Hz
Supplementary Data 17. Figure S7d_0.5Hz
Supplementary Data 18. Figure S7d_1Hz
Supplementary Data 19. Figure S7e
Supplementary Data 20. Figure S8
