# Supplementary material for: Pantethine ameliorates dilated cardiomyopathy features in PPCS deficiency disorder in patients and cell line models
Source: Commun Med (Lond). 2025 Jul 31;5:323. doi: 10.1038/s43856-025-01017-z (PMC12313872; doi:10.1038/s43856-025-01017-z)
Supplement: Supplementary file 23 — Reporting Summary [file 43856_2025_1017_MOESM23_ESM.pdf]

Corresponding author(s): Arcangela Iuso; Alessandra Moretti

Last updated by author(s): 29/04/2025

Reporting Summary

Nature Portfolio wishes to improve the reproducibility of the work that we publish. This form provides structure for consistency and transparency in reporting. For further information on Nature Portfolio policies, see our Editorial Policies and the Editorial Policy Checklist.

Statistics

For all statistical analyses, confirm that the following items are present in the figure legend, table legend, main text, or Methods section.

n/a	Confirmed	
☐	☒	The exact sample size (*n*) for each experimental group/condition, given as a discrete number and unit of measurement
☐	☒	A statement on whether measurements were taken from distinct samples or whether the same sample was measured repeatedly
☐	☒	The statistical test(s) used AND whether they are one- or two-sided<br>*Only common tests should be described solely by name; describe more complex techniques in the Methods section.*
☒	☐	A description of all covariates tested
☐	☒	A description of any assumptions or corrections, such as tests of normality and adjustment for multiple comparisons
☐	☒	A full description of the statistical parameters including central tendency (e.g. means) or other basic estimates (e.g. regression coefficient) AND variation (e.g. standard deviation) or associated estimates of uncertainty (e.g. confidence intervals)
☒	☐	For null hypothesis testing, the test statistic (e.g. F, t, r) with confidence intervals, effect sizes, degrees of freedom and P value noted<br>*Give P values as exact values whenever suitable.*
☒	☐	For Bayesian analysis, information on the choice of priors and Markov chain Monte Carlo settings
☒	☐	For hierarchical and complex designs, identification of the appropriate level for tests and full reporting of outcomes
☒	☐	Estimates of effect sizes (e.g. Cohen's d, Pearson's r), indicating how they were calculated

Our web collection on statistics for biologists contains articles on many of the points above.

Software and code

Policy information about availability of computer code

Data collection	Excel, Prism
Data analysis	Excel, Prism

For manuscripts utilizing custom algorithms or software that are central to the research but not yet described in published literature, software must be made available to editors and reviewers. We strongly encourage code deposition in a community repository (e.g. GitHub). See the Nature Portfolio guidelines for submitting code & software for further information.

Data

Policy information about availability of data

All manuscripts must include a data availability statement. This statement should provide the following information, where applicable:

- Accession codes, unique identifiers, or web links for publicly available datasets
- A description of any restrictions on data availability
- For clinical datasets or third party data, please ensure that the statement adheres to our policy

The authors confirm that the data supporting the findings of this study are available within the article and its supplementary materials.

Human research participants

Policy information about studies involving human research participants and Sex and Gender in Research.

Reporting on sex and gender	Our inclusion criteria encompassed patients of all ages and geneders with genetically confirmed PPCS mutations, regardless of their current clinical status or previous treatments. This approach allowed us to capture a diverse range of disease presentations and progression stages. We also ensured diversity in experimental samples by selecting various cell lines. The author list of this paper includes contributors from the research location who participated in data collection, design, analysis, and/or interpretation of the work.
Population characteristics	Our inclusion criteria encompassed patients of all ages and genders with genetically confirmed PPCS mutations, regardless of their current clinical status or previous treatments. This approach allowed us to capture a diverse range of disease presentations and progression stages. We also ensured diversity in experimental samples by selecting various cell lines. The author list of this paper includes contributors from the research location who participated in data collection, design, analysis, and/or interpretation of the work.
Recruitment	Patients for this study were identified through a comprehensive review of medical records at participating tertiary care centers specializing in rare metabolic disorders and cardiology. We focused on individuals with confirmed genetic mutations in the PPCS gene, which is associated with the condition under investigation. Potential participants were initially screened based on their clinical presentation, family history, and genetic test results. Once identified, these patients and their families were approached by their treating physicians, who provided information about the study. We explained the study's objectives, procedures, potential risks, and benefits in detail. A list of study participants, clinical features and information on pantethine intervention is provided in Tables S1.
Ethics oversight	Informed consent was obtained from all adult participants, while for minors, assent was obtained along with parental consent. The study was conducted in accordance with the principles embodied in the Declaration of Helsinki and approved by the Institutional Review Boards of the Children's Hospital of Philadelphia (16-013278), Sheba Medical Center (SMC615819) and Hadassah University Hospital (0151-20-HMO) ensuring ethical standards were maintained throughout the recruitment process. The use of pantethine for the patients was approved as a compassionate treatment (29C) by the Israeli Ministry of Health.<br>Generation of iPSCS from patient fibroblasts and differentiation to cardiac cells was approved by the Ethics Committee of the Technical University of Munich (2022-220-S-NP).<br>Fresh pig hearts for the generation of porcine extracellular matrix were obtained from Munich slaughterhouse as a waste from a licensed abattoir producing meat for consumption operating under strict government protocols. Therefore, the ethical approval of fresh pig hearts was not required.

Note that full information on the approval of the study protocol must also be provided in the manuscript.

Field-specific reporting

Please select the one below that is the best fit for your research. If you are not sure, read the appropriate sections before making your selection.

☒ Life sciences ☐ Behavioural & social sciences ☐ Ecological, evolutionary & environmental sciences

For a reference copy of the document with all sections, see nature.com/documents/nr-reporting-summary-flat.pdf

Life sciences study design

All studies must disclose on these points even when the disclosure is negative.

Sample size	Not applicable; Resource constraints to recruit a large number of cases due to the rarity of the disease
Data exclusions	Not applicable
Replication	All cell biology experiments were successfully replicated
Randomization	Not relevant to the study because of the rarity of the disease
Blinding	Not relevant to the study because of the rarity of the disease

Reporting for specific materials, systems and methods

We require information from authors about some types of materials, experimental systems and methods used in many studies. Here, indicate whether each material, system or method listed is relevant to your study. If you are not sure if a list item applies to your research, read the appropriate section before selecting a response.

Materials & experimental systems

n/a	Involved in the study
☐	☒ Antibodies
☐	☒ Eukaryotic cell lines
☒	☐ Palaeontology and archaeology
☐	☒ Animals and other organisms
☐	☒ Clinical data
☒	☐ Dual use research of concern

Methods

n/a	Involved in the study
☒	☐ ChIP-seq
☐	☒ Flow cytometry
☒	☐ MRI-based neuroimaging

Antibodies

Antibodies used	PPCS (ab140626, Abcam); Tubulin (T5168, Sigma); Actin (A5441, Sigma); TOM20 (sc-11415, Santa Cruz); GAPDH (ab110305, Abcam); Troponin (cTnT, Thermo Fischer Scientific, MA5-12960; Abcam, ab92546), α-actinin (Sigma-Aldrich, A7811), ISL1 (DSHB, 39.4D5); PPCS (Thermo Fisher Scientific, PA5-95630); Phalloidin (Thermo Fisher Scientific, A12379); HRP-conjugated secondary antibodies (111-036-045; 115-036-062, Jackson Immunoresearch Laboratories); AF647 anti-rabbit (ab150079, Abcam); AF568 anti-mouse (A11004, Invitrogen)
Validation	Western Blotting: PPCS (ab140626, Abcam); Tubulin (T5168, Sigma); Actin (A5441, Sigma); HRP-conjugated secondary antibodies (111-036-045; 115-036-062, Jackson Immunoresearch Laboratories) Immunocytochemistry(immunofluorescence: TOM20 (sc-11415, Santa Cruz); GAPDH (ab110305, Abcam); Troponin (cTnT, Thermo Fischer Scientific, MA5-12960; Abcam, ab92546), α-actinin (Sigma-Aldrich, A7811), ISL1 (DSHB, 39.4D5); PPCS (Thermo Fisher Scientific, PA5-95630); Phalloidin (Thermo Fisher Scientific, A12379); AF647 anti-rabbit (ab150079, Abcam); AF568 anti-mouse (A11004, Invitrogen);

Eukaryotic cell lines

Policy information about cell lines and Sex and Gender in Research

Cell line source(s)	NHDF, #CC-2509, Lonza; HeLa cells (#CCL-2, ATCC); primary skin fibroblasts from patients; iPSCs from patients
Authentication	Vendor authentication for NHDF and HeLa; Sanger sequencing valdation for primary skin fibroblasts; STR and Sanger sequencing validation for iPSCs from patients
Mycoplasma contamination	All cell lines were free from mycoplasma contamination
Commonly misidentified lines (See ICLAC register)	*Name any commonly misidentified cell lines used in the study and provide a rationale for their use.*

Animals and other research organisms

Policy information about studies involving animals; ARRIVE guidelines recommended for reporting animal research, and Sex and Gender in Research

Laboratory animals	Not applicable
Wild animals	*Provide details on animals observed in or captured in the field; report species and age where possible. Describe how animals were caught and transported and what happened to captive animals after the study (if killed, explain why and describe method; if released, say where and when) OR state that the study did not involve wild animals.*
Reporting on sex	*Indicate if findings apply to only one sex; describe whether sex was considered in study design, methods used for assigning sex. Provide data disaggregated for sex where this information has been collected in the source data as appropriate; provide overall numbers in this Reporting Summary. Please state if this information has not been collected. Report sex-based analyses where performed, justify reasons for lack of sex-based analysis.*
Field-collected samples	*For laboratory work with field-collected samples, describe all relevant parameters such as housing, maintenance, temperature, photoperiod and end-of-experiment protocol OR state that the study did not involve samples collected from the field.*
Ethics oversight	*Identify the organization(s) that approved or provided guidance on the study protocol, OR state that no ethical approval or guidance was required and explain why not.*

Note that full information on the approval of the study protocol must also be provided in the manuscript.

Clinical data

Policy information about clinical studies

All manuscripts should comply with the ICMJE guidelines for publication of clinical research and a completed CONSORT checklist must be included with all submissions.

Clinical trial registration	Not applicable
Study protocol	*Note where the full trial protocol can be accessed OR if not available, explain why.*
Data collection	*Describe the settings and locales of data collection, noting the time periods of recruitment and data collection.*
Outcomes	*Describe how you pre-defined primary and secondary outcome measures and how you assessed these measures.*

Flow Cytometry

Plots

Confirm that:

☒ The axis labels state the marker and fluorochrome used (e.g. CD4-FITC).

☒ The axis scales are clearly visible. Include numbers along axes only for bottom left plot of group (a 'group' is an analysis of identical markers).

☒ All plots are contour plots with outliers or pseudocolor plots.

☒ A numerical value for number of cells or percentage (with statistics) is provided.

Methodology

Sample preparation	For flow cytometry analysis of cTnT, iPSC-CMs at day 15 of differentiation were dissociated with papain and 5x106 cells were fixed in 4% PFA for 15 min at RT. The samples were permeabilized with 0.25% Triton X-100 in DPBS+/+ for 15 minutes and blocked with 10% FBS in PBST for 1 hour at RT. Afterwards, the cells were incubated with primary antibody for cTnT or IgG isotype control in 1% FBS in PBST on a shaker overnight at 4°C. After three times washing with PBST, fluorescent dye-conjugated anti-rabbit secondary antibody was incubated for 1 hour at RT.
Instrument	The cells were measured on the Gallios flow cytometer (Beckman Coulter, Germany)
Software	Data were analyzed using Kaluza software version 1.2 (Beckman Coulter)
Cell population abundance	Equivalent CM populations were obtained from control iPSCs and iPSCs from patients 95595 and 103596 (around 80% cTnT+ cells)
Gating strategy	SSC

☒ Tick this box to confirm that a figure exemplifying the gating strategy is provided in the Supplementary Information.
